# Diversity and Distribution of Harmful Algal Bloom Species from Seamount to Coastal Waters in the South China Sea

**DOI:** 10.1128/spectrum.04169-22

**Published:** 2023-02-23

**Authors:** Hailong Huang, Shuaishuai Xu, Shuangqing Li, Xinwei Wang, Kangli Guo, Rongman Yan, Wei Xie, Kedong Yin, Shengwei Hou, Haibo Jiang

**Affiliations:** a School of Marine Sciences, Ningbo University, Ningbo, Zhejiang, China; b Southern Marine Science and Engineering Guangdong Laboratory (Zhuhai), China; c School of Marine Sciences, Sun Yat-sen University, Guangzhou, China; d Department of Ocean Science and Engineering, Southern University of Science and Technology, Shenzhen, China; e College of Life Science and Technology, Jinan University, Guangzhou, China; f State Key Laboratory for Marine Environmental Science, Institute of Marine Microbes and Ecospheres, Xiamen University, Xiamen, China; University of Mississippi

**Keywords:** South China Sea, SCS, seamounts, harmful algal blooms, metabarcoding analysis, amplicon sequence variants, ASV

## Abstract

Mount Xianbei is one of the largest shallow seamounts located in the middle of the South China Sea (SCS), which might play a role in shaping the biodiversity of surrounding continental coastal waters, particularly the diversity of phytoplankton species causing frequent harmful algal blooms (HABs) in northern SCS. However, the diversity, composition, and distribution of phytoplankton species in the seamount regions of Xianbei remain largely unexplored. In this study, samples around and outside the seamount regions were collected during a late summer cruise of 2021 to test whether seamounts play a role in HAB species propagation. In total, we identified 19 HAB species across all samples using the ASV-based DNA metabarcoding approach, 6 of which had not been reported previously in the SCS, suggesting a diverse HAB species in the SCS. Specifically, 16 HAB species were found in the seamount region of Xianbei, and 5 of them were also found in the coastal waters, indicating a close connection between seamount and coastal waters. This study was the first attempt to explore HAB species’ spatial diversity and vertical distribution in the seamount region of Xianbei at single-nucleotide resolution, which provides a novel explanation for the coastal HAB occurrence in the northern SCS.

**IMPORTANCE** There are a number of seamounts under the water of the South China Sea (SCS). The seamounts might play a role in shaping the biodiversity of surrounding continental coastal waters. However, there is no direct evidence revealing the relationship of the biodiversity of phytoplankton between seamounts and coastal waters in the SCS, especially those species having the potential to form harmful algal blooms (HABs). Some HAB species might proliferate in certain geographic locations, while others may be broadly distributed across oceanic provinces. In this study, we provided a detailed analysis of phytoplankton composition and molecular detection of HAB species from seamount to coastal waters in the SCS, which suggested a strong interaction in the HAB species between the two areas. This finding provides new insights into the diversity and distribution of HABs in seamounts and their role in shaping the composition and the occurrence of HABs in coastal water.

## INTRODUCTION

Marine harmful algal blooms (HABs) are destructive biological events caused by rapid production and accumulation (retention, physical transport, behavior) of microalgae (1). In the past few decades, both the frequency and duration of HABs have been increasing worldwide, posing a major threat to human health, economic stability, and marine ecosystem functioning (2, 3). Some HAB species might proliferate in certain geographic locations, while others may be broadly distributed across oceanic provinces (4–6). There is an increasing number of rare and novel HAB species detected outside the geographical area where they were first identified, and outbreaks caused by the same HAB species may be geographically distant (7). For instance, the picoplanktonic (2 to 3 μm) pelagophyte Aureococcus anophagefferens first appeared during a “brown tide” event in coastal bays in the northeastern United States in 1985 (8) but subsequently occurred in Saldanha Bay, South Africa in 1997 (9) and in the coastal area of Qinhuangdao, China in 2009 (10). *A. anophagefferens* was detected in the ballast water of local watercraft, which was regarded as the vector of *A. anophagefferens* dispersion. However, in a recent study, Tang et al. (11) reported that *A. anophagefferens* was not recently introduced but rather an indigenous species in the northern South China Sea, suggesting *A. anophagefferens* might have a cosmopolitan distribution globally regardless of maritime activities. Similarly, the toxin-producing dinoflagellate Stoeckeria algicida was first discovered off the south coast of Korea in 2005 and was later discovered in Liaodong bay in the Bohai Sea in 2014, presumably introduced by ocean currents or shipping (12). Therefore, it is essential to study the diversity, community dynamics, and geographical distribution of HAB species across ocean basins to trace the origin and propagation of HAB species.

The South China Sea (SCS) is the largest marginal sea in the western tropical Pacific Ocean and is characterized by complex physicochemical environments (13). The coastal waters of the SCS, especially the Pearl River Estuary, are suffering from the high incidence of HABs (14). There are many seamounts scattered across the vast abyssal plain of the SCS. Xianbei is a shallow seamount located in the central part of the SCS with a height of 3,786 m and a peak 208 m below the surface sea, close to the euphotic zone. Seamounts provide special habitats for marine organisms, which have unique biological characteristics due to their specific geographical characteristics and hydrological conditions (15). Previous studies have shown that physical processes such as Taylor cones in shallow seamounts will form a “seamount effect,” which brings rich nutrients in deep water into the photic zone through upwelling, thus promoting phytoplankton growth and increasing primary and secondary productivity (16–18). In addition, seamounts may form shore-like habitats where the phytoplankton community structure may differ from that of the adjacent open ocean (19). The phytoplankton diversity and distribution patterns in the SCS, particularly in seamount regions, are not well understood. In addition, how HAB species differ in seamount regions from the Pearl River Estuary is not clear.

Studies on phytoplankton and HAB species in the SCS originated in the 1870s and have accumulated abundant data; these studies heavily relied on the morphological analysis of algal species and mainly involved classifying, identifying, and counting phytoplankton cells under a microscope on the basis of morphological characteristics. Traditional methods are not only tedious but less accurate in species identification solely based on morphology, compared to molecular biology-based methods. Particularly, DNA metabarcoding methods based on molecular marker amplification and the high-throughput sequencing approach developed quickly in recent years (20–22), which have been proven to be powerful in analyzing phytoplankton diversity and community composition. The metabarcoding approach has been applied in global ocean microbial surveys, including the Tara Oceans Expedition (23) and the Ocean Sampling Day (24). Previously, Xu et al. (7) have shown that there are large numbers of phytoplankton and HAB species in the Western Pacific seamount regions, while only a few studies reported that HAB species were also present in seamount regions of the SCS. What are the HAB species and how are they distributed in the SCS and in coastal waters remain unclear.

More recently, McNichol et al. (25) compared and evaluated the coverage of primers for different rRNA subunits by using the metagenomic data of global marine bacteria, archaea, and eukaryotes. The finding revealed that the expected performance of primer sets could be improved with minor modifications, pointing toward a nearly completely universal primer set (515Y/926R) that could accurately quantify biogeochemically important taxa in ecosystems ranging from the deep sea to the surface. Compared with the universal primers for the variable regions of 18S rDNA (V1 to V9) commonly used in eukaryotic phytoplankton, this primer set has a higher amplification efficiency and can obtain more complete species information, including cyanobacteria. Furthermore, most metabarcoding-based analyses were performed using operational taxonomic units (OTU) clustering, but 97 to 98% sequence similarity clustering reduced species resolution, resulting in a large number of species and important strains being ignored in the survey (26–28). Meanwhile, new methods have been developed that resolve amplicon sequence variants (ASVs) from Illumina-scale amplicon data without imposing the arbitrary dissimilarity thresholds that define molecular OTUs (29, 30). The ASV-based method has demonstrated sensitivity and specificity as good or better than the OTU-based method, while the ASV-based method is explicitly intended to replace OTU-based analysis (30, 31). This approach has been proven to be effective for analyzing protistan diversity and distributions in nature (22) but has only recently been applied to HAB species surveys (26, 27, 32, 33).

Based on previous studies, we hypothesize that phytoplanktonic biodiversity and community composition in the seamount region of Xianbei may be different from surrounding and coastal waters, and seamount might host diverse HAB species that could potentially seeding coastal HABs. To test this hypothesis, we applied an ASV-based metabarcoding approach to analyze amplicon sequences recovered from 61 water samples collected in the SCS, during August and September in 2021. Our main objectives of this study were to (i) compare phytoplankton community composition, richness, diversity, and relative abundance (including eukaryotic algae and cyanobacteria) in the samples from seamount region of Xianbei (XB), Xisha (XS), and Dongsha (DS) of SCS; (ii) investigate HAB species composition and distribution in the seamount region of Xianbei; (iii) explore the dominant HAB species and their correlations with environmental factors in seamount region of Xianbei along depth gradients; and (iv) evaluate the composition and similarity of HAB species between coastal waters and the seamount region of Xianbei in the SCS.

## RESULTS

### High-throughput sequencing data statistics.

**FIG 1:**
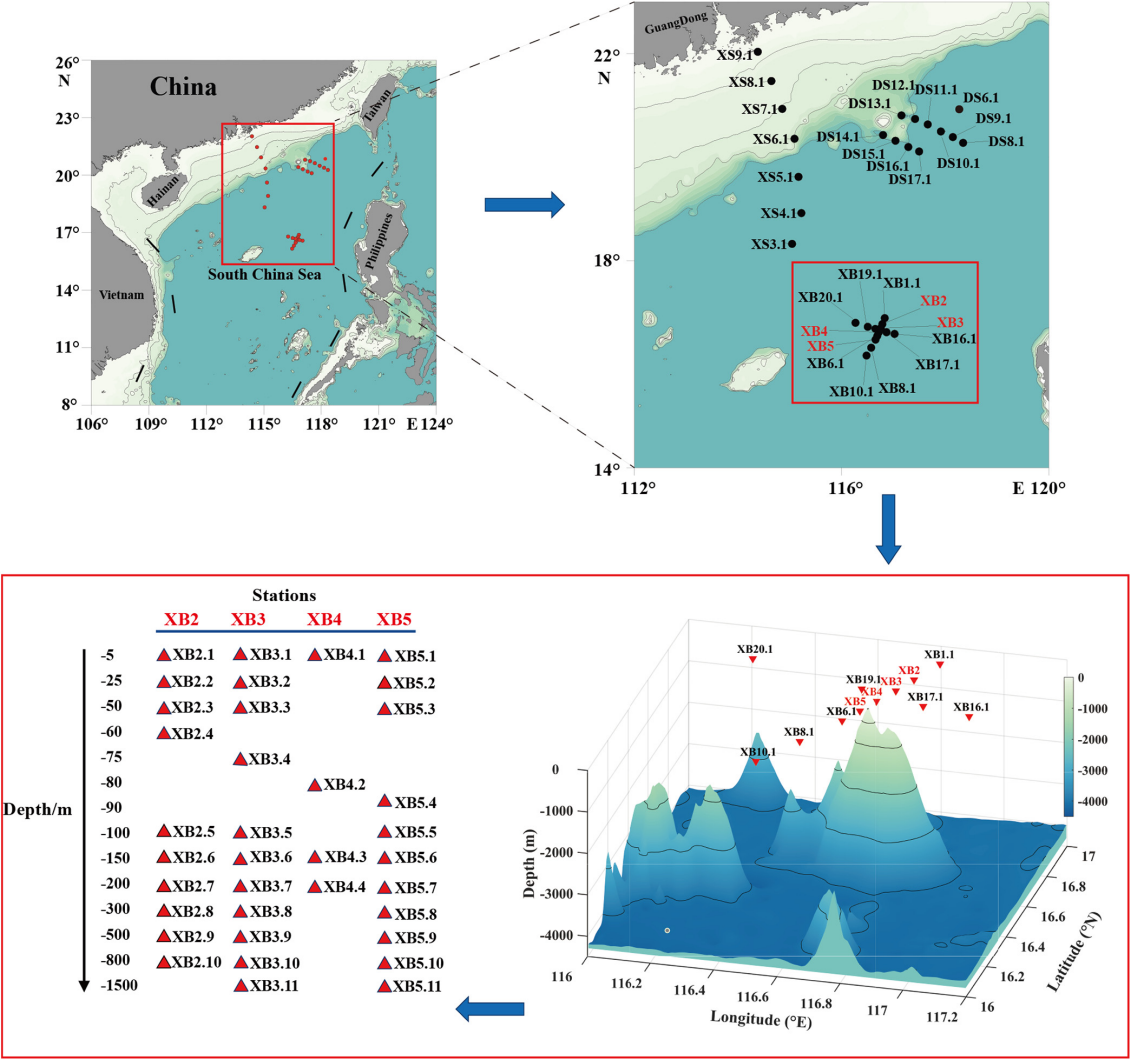
Geographic map of the 30 sampling sites in the South China Sea (SCS). Solid dots represent sampling sites. The dots are profile locations labeled with code numbers The darker blue colors represent the deeper waters.

A total of ~22,795,694 raw rRNA gene reads were obtained from all 61 samples at 30 sampling sites in the SCS from August to September of 2021 (Fig. 1). After filtering the low-quality reads, trimming the adapters and barcodes, and removing chimeras using the DADA2 pipeline, 3,251 ASVs were obtained from all water samples with an average length of 373 nucleotides. Of these, 2,776 ASVs were supported by at least 2 reads and detected in at least 1 sample. In this study, we focused on the analysis of 939 ASVs that were annotated as phytoplankton species. The rarefaction analysis indicated near saturation, suggesting sufficient sequencing depths (Fig. S1 in the supplemental material).

**FIG 2:**
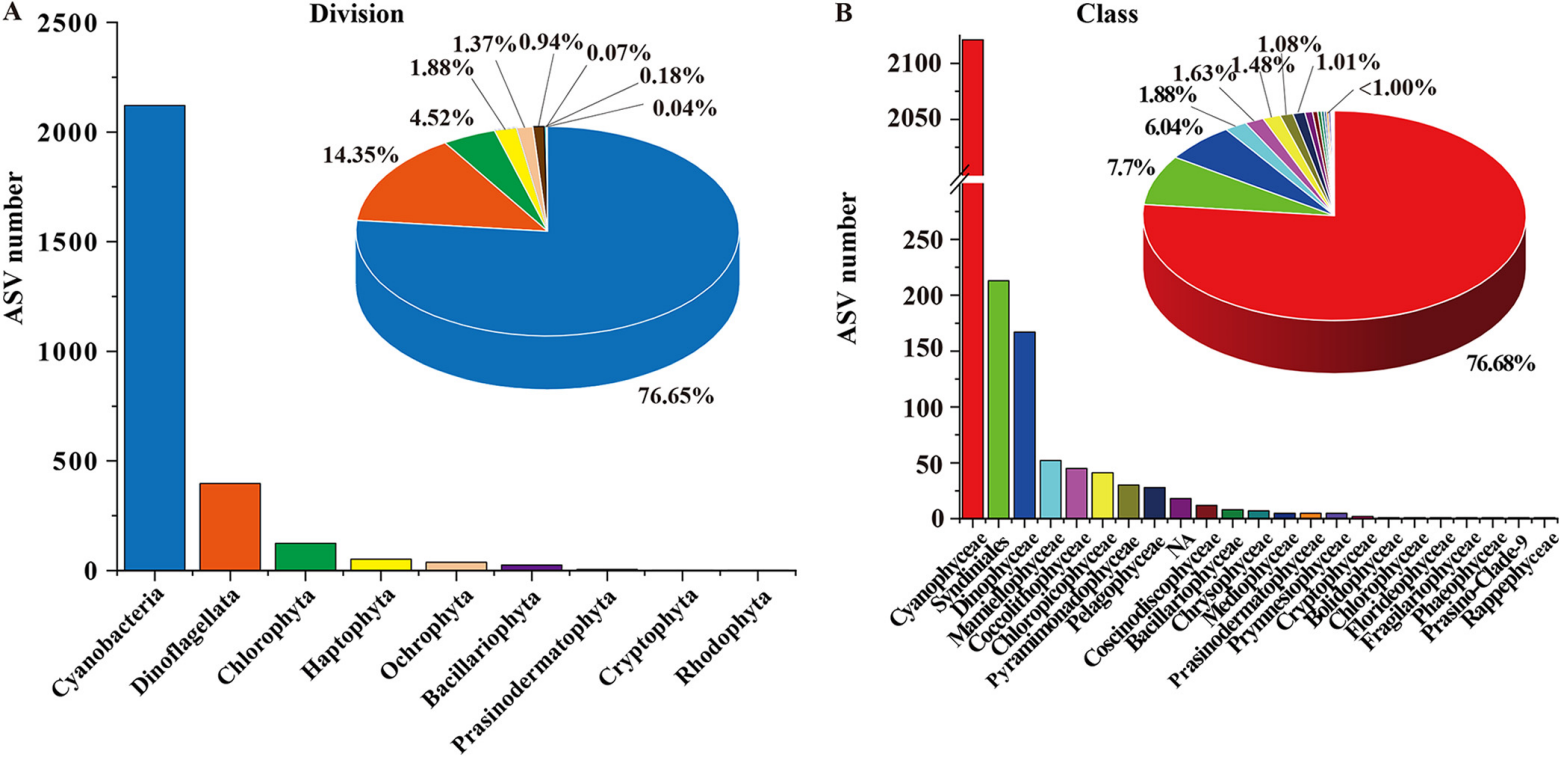
Overview of assemblages of phytoplankton in the South China Sea. Richness and relative abundance of phytoplankton at division (A) and class (B) taxonomic levels.

Among all 2,776 ASVs annotated as phytoplankton, the richness of the Cyanobacteria division was the highest (2,121 ASVs, accounting for 76.65%), followed by Dinoflagellata (406 ASVs, 14.35%), Chlorophyta (125 ASVs, 4.52%), Haptophyta (52 ASVs, 1.88%), Ochrophyta (38 ASVs, 1.37%), Bacillariophyta (26 ASVs, 0.94%), Prasinodermatophyta (5 ASVs, 0.18%), Cryptophyta (2 ASVs, 0.07%), and Rhodophyta (1 ASV, 0.04%) (Fig. 2A). At the class level, 22 classes were identified (Fig. 2B). The richness of Cyanophyceae class (2121 ASVs, 76.68%) was the highest, followed by Syndiniales (213 ASVs, 7.70%), Dinophyceae (167 ASVs, 6.04%), Mamiellophyceae (52 ASVs, 1.88%), Coccolithophyceae (45 ASVs, 1.63%), Chloropicophyceae (41ASVs, 1.48%), Pyramimonadophyceae (30 ASVs, 1.08%), Pelagophyceae (28 ASVs, 1.01%), and others.

### Diversity analysis of phytoplankton in the SCS.

**FIG 3:**
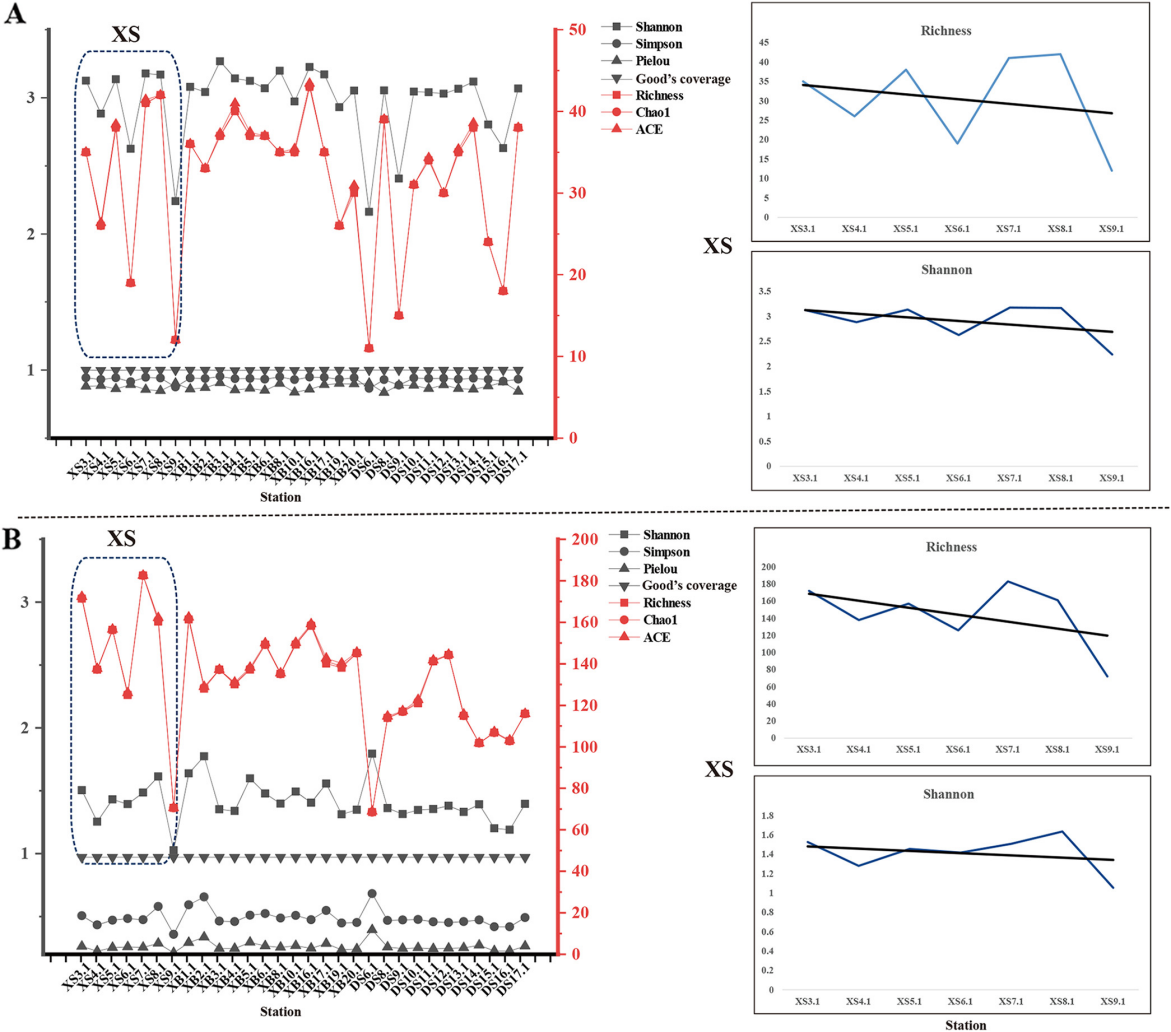
Alpha diversity indexes based on the normalized data for the surface water samples from Xisha (XS), Dongsha (DS), and Xianbei (XB) three studied sea regions, in the eukaryotic algae survey (A) and cyanobacteria survey (B). To get a better view of the trend, the indexes of Richness and Shannon were displayed with magnification. The indexes were generated with the ggplot2 package.

Most of the rarefaction curves for the 61 samples tended to reach saturation (Fig. S1), and the Good’s coverage indexes ranged from 0.99 to 1.00, suggesting sufficient sequencing depth for this study. To explore the richness and evenness of phytoplankton at all sampling sites, alpha diversity indexes were calculated for surface water samples from XS, DS, and XB (Fig. 3) (Table S1 and S2). The Richness and Shannon indexes of eukaryotic algae (Fig. 3A) and cyanobacteria (Fig. 3B) fluctuated within a certain range, showing that XB16.1 had the largest richness and XS9.1 and DS6.1 had the smallest richness. In particular, the Richness and Shannon indexes of samples in XS and DS were relatively lower than that of samples in XB. In addition, the Richness and Shannon indexes of the XS samples from the sampling sites XS3.1 in the open sea to XS9.1 near the Pearl River Estuary showed a slight downward trend. Comparative diversity analysis of phytoplankton ASVs in all sampling sites revealed higher diversity of eukaryotic algae than cyanobacteria (Fig. 3).

**FIG 4:**
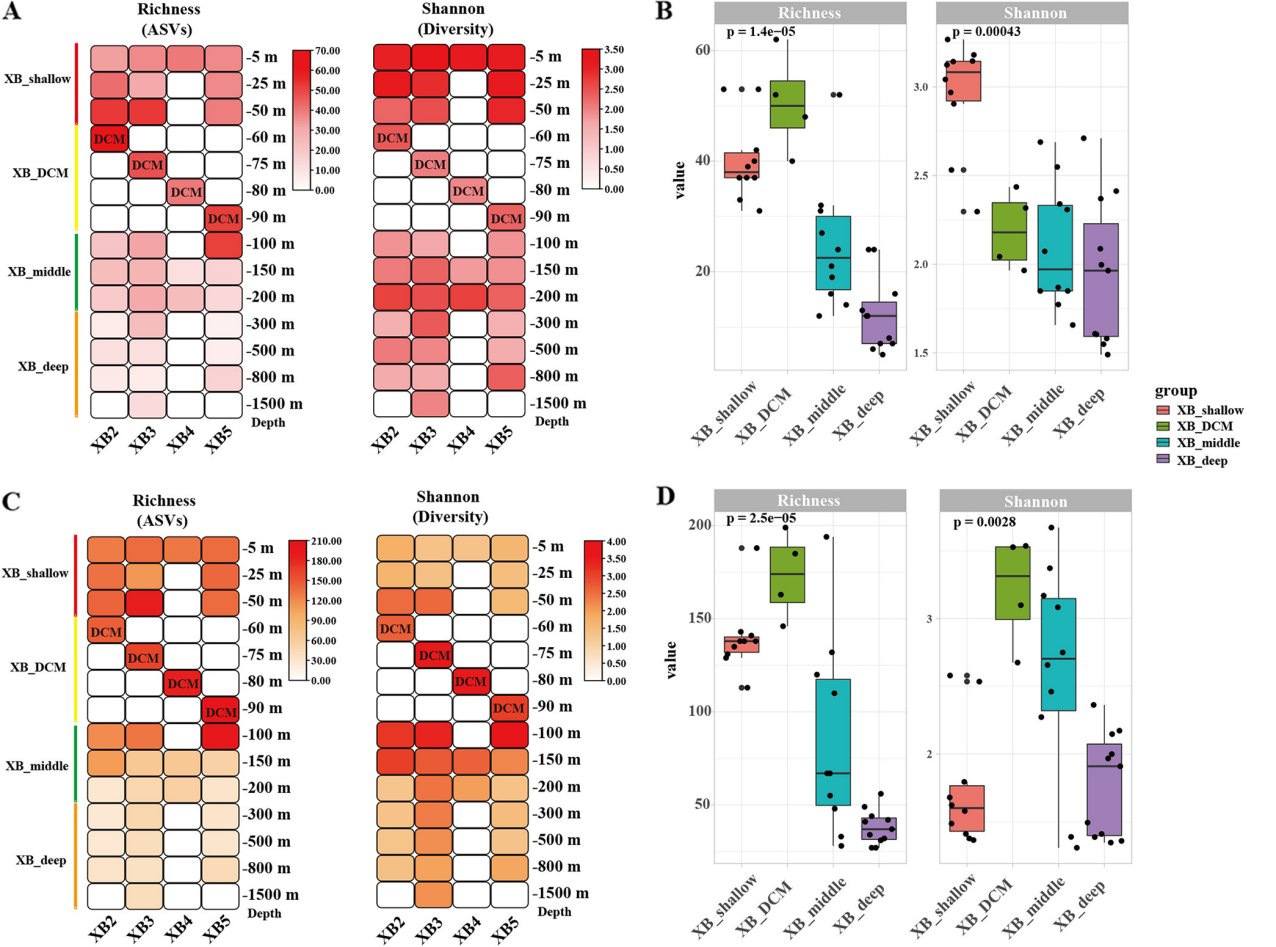
Alpha-diversity indexes of the phytoplankton from four groups along the depth gradients in XB2 to XB5 from the seamount region of Xianbei, in the eukaryotic algae survey (A and B) and cyanobacteria survey (C and D). Four groups: XB_shallow (−5, −25, and −50 m), XB_DCM (−60, −75, −80, and −90 m), XB_middle (−100, −150, and −200 m), and XB_deep (−300, −500, −800, and −1,500 m).

To reveal the differences of alpha diversity along the depth gradients in the seamount region of Xianbei, samples were categorized into four groups: XB_shallow (−5, −25, and −50 m), XB_DCM (−60, −75, −80, and −90 m), XB_middle (−100, −150, and −200 m), and XB_deep (−300, −500, −800, and −1,500 m). In the eukaryotic algae survey (Fig. 4A and B), analysis of the alpha-diversity metrics (Richness and the Shannon index) indicated that the Richness index of the XB_DCM water group was relatively higher than other groups. However, the Shannon index of the XB_shallow water group was the highest in four groups. In the cyanobacteria survey (Fig. 4C and D), the results of alpha diversity also showed the Richness index of the XB_DCM was the highest between the XB_shallow water group and the XB_middle, XB_DCM, and XB_deep groups. In addition, the Shannon index of the XB_shallow water group was lowest than other groups. Notably, the trend of Shannon diversity of eukaryotic algae was opposite to that of cyanobacteria across sampling depth.

### Phytoplankton community composition and characteristics.

The composition and relative phytoplankton abundance at different sampling sites were compared at division, class, genus, and species levels for eukaryotic algae and cyanobacteria, respectively (Fig. S2 and S3). At the division level, we found that Ochrophyta (orange) and Chlorphyta (blue) were the dominant eukaryotic algae at sampling sites XB2.3, XB2.4, XB3.3, XB4.2, XB5.4, and XB5.5 from the seamount region of Xianbei (Fig. S2A and B). At the class level, different classes of eukaryotic algae also showed uneven distribution patterns across sampling sites, with Pelagophyceae and Mamiellophyceae being the dominant groups (Fig. S2C and D). The dominant groups of phytoplankton at the division and class levels from the eukaryotic algae survey were mainly from sampling sites in the seamount region of Xianbei. In addition, *Prochlorococcus* and *Prochlorococcus* MIT9313 were the most dominant genus (Fig. S3A and B) and species (Fig. S3C and D), followed by *Synechococcus* and *Synechococcus* PCC6307 in the cyanobacteria survey, respectively. In summary, the distribution of richness and relative abundance of eukaryotic algae presented a significant geographical distribution advantage in the seamount region of Xianbei. In contrast, *Prochlorococcus* of cyanobacteria was dominant at almost all sampling sites.

### HAB species composition and distribution in the seamount region of Xianbei.

**FIG 5:**
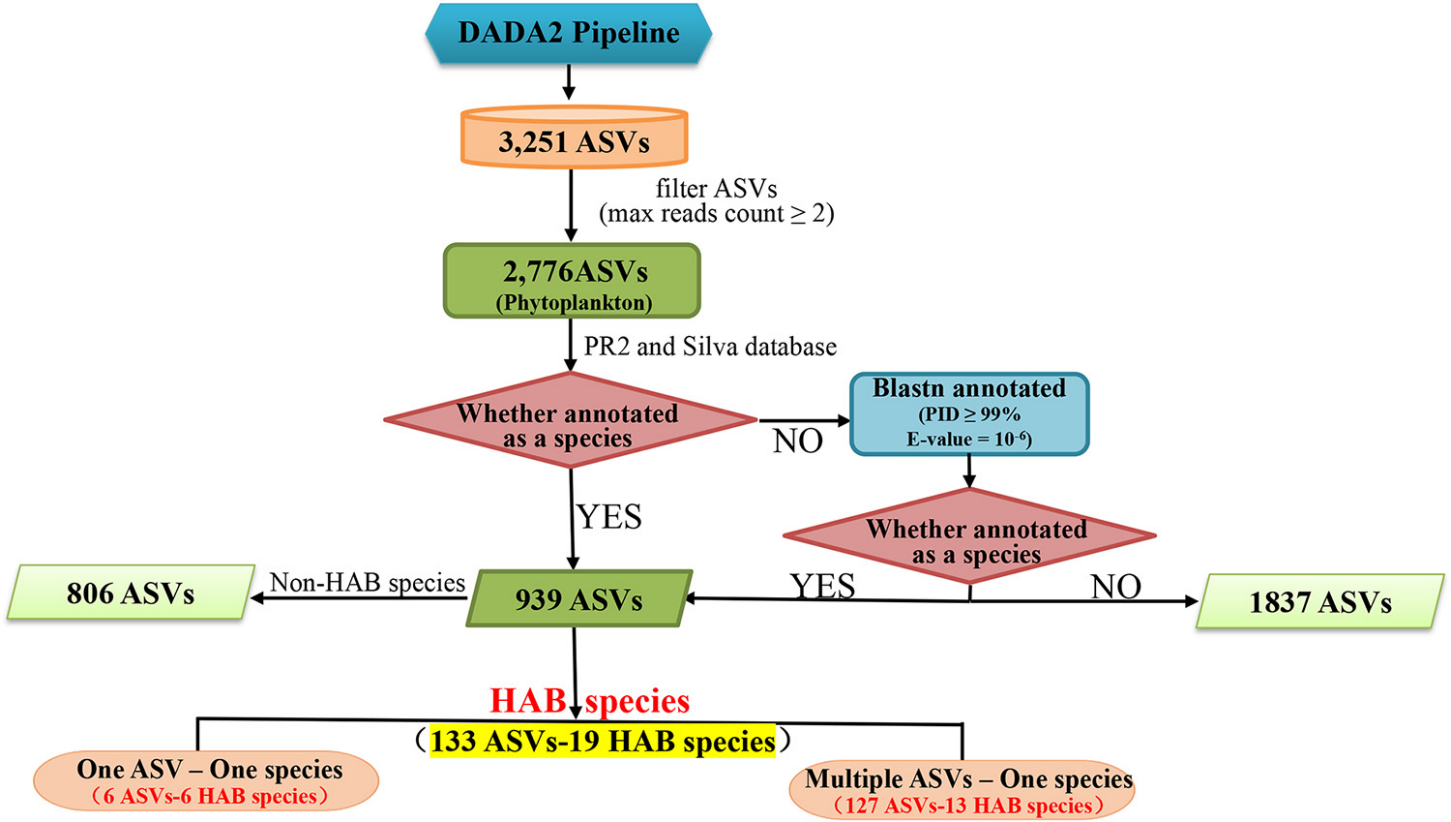
A flowchart describing the ASVs annotation and processing procedure for HAB species.

Among 2,776 phytoplankton ASVs (including eukaryotic algae and cyanobacteria), 939 (33.8%) could be annotated to specific species, while 1,837 (66.2%) could not (Fig. 5), suggesting phytoplankton diversity in the SCS is largely untapped. Among these 939 ASVs, 133 ASVs were annotated to 19 HAB species with one-to-one and many-to-one ASV-species relationships (Table 1). Specifically, 6 ASVs could be assigned to 6 HAB species with one-to-one ASV-species relationships, and 127 ASVs could be assigned to 13 HAB species. The presence of multiple ASVs corresponding to one species suggested that these species might have high levels of genetic diversity, some of which might represent previously unreported cryptic species. Among these 19 identified HAB species, 6 HAB species have not been reported previously in the SCS, indicating the discovery power of ASV-based metabarcoding analysis. These six HAB species are Gymnodinium aureolum (Dinoflagellata), Heterocapsa rotundata (Dinoflagellata), Karlodinium veneficum (Dinoflagellata), Prymnesium polylepis (Haptophyta), Thalassiosira diporocyclus (Bacillariophyta), and Pyramimonas parkeae (Chlorophyta).

**TABLE 1:** The annotation information of HAB species and their corresponding ASVs^*a*^

ASV ID	Division	Class	Species	Group
ASV234	Dinoflagellata	Dinophyceae	Gonyaulax polygramma* ^*b*^	1 vs 1
ASV628	Dinoflagellata	Dinophyceae	Alexandrium leei*	1 vs 1
ASV63	Dinoflagellata	Dinophyceae	Katodinium glaucum*	1 vs 1
ASV727	Bacillariophyta	Coscinodiscophyceae	Thalassiosira diporocyclus**	1 vs 1
ASV966	Bacillariophyta	Bacillariophyceae	Pseudo-nitzschia multiseries*	1 vs 1
ASV2465	Chlorophyta	Pyramimonadophyceae	Pyramimonas parkeae** ^*c*^	1 vs 1
ASV750	Ochrophyta	Pelagophyceae	Aureococcus anophagefferens*	1 vs m
ASV979	Ochrophyta	Pelagophyceae	*Aureococcus anophagefferens*	1 vs m
ASV1163	Ochrophyta	Pelagophyceae	*Aureococcus anophagefferens*	1 vs m
ASV1177	Ochrophyta	Pelagophyceae	*Aureococcus anophagefferens*	1 vs m
ASV1251	Ochrophyta	Pelagophyceae	*Aureococcus anophagefferens*	1 vs m
ASV1313	Ochrophyta	Pelagophyceae	*Aureococcus anophagefferens*	1 vs m
ASV1525	Ochrophyta	Pelagophyceae	*Aureococcus anophagefferens*	1 vs m
ASV1562	Ochrophyta	Pelagophyceae	*Aureococcus anophagefferens*	1 vs m
ASV1578	Ochrophyta	Pelagophyceae	*Aureococcus anophagefferens*	1 vs m
ASV1665	Ochrophyta	Pelagophyceae	*Aureococcus anophagefferens*	1 vs m
ASV1988	Ochrophyta	Pelagophyceae	*Aureococcus anophagefferens*	1 vs m
ASV2004	Ochrophyta	Pelagophyceae	*Aureococcus anophagefferens*	1 vs m
ASV2031	Ochrophyta	Pelagophyceae	*Aureococcus anophagefferens*	1 vs m
ASV2035	Ochrophyta	Pelagophyceae	*Aureococcus anophagefferens*	1 vs m
ASV2036	Ochrophyta	Pelagophyceae	*Aureococcus anophagefferens*	1 vs m
ASV2255	Ochrophyta	Pelagophyceae	*Aureococcus anophagefferens*	1 vs m
ASV2322	Ochrophyta	Pelagophyceae	*Aureococcus anophagefferens*	1 vs m
ASV2390	Ochrophyta	Pelagophyceae	*Aureococcus anophagefferens*	1 vs m
ASV2484	Ochrophyta	Pelagophyceae	*Aureococcus anophagefferens*	1 vs m
ASV2498	Ochrophyta	Pelagophyceae	*Aureococcus anophagefferens*	1 vs m
ASV2569	Ochrophyta	Pelagophyceae	*Aureococcus anophagefferens*	1 vs m
ASV2584	Ochrophyta	Pelagophyceae	*Aureococcus anophagefferens*	1 vs m
ASV2701	Ochrophyta	Pelagophyceae	*Aureococcus anophagefferens*	1 vs m
ASV2813	Ochrophyta	Pelagophyceae	*Aureococcus anophagefferens*	1 vs m
ASV2967	Ochrophyta	Pelagophyceae	*Aureococcus anophagefferens*	1 vs m
ASV3028	Ochrophyta	Pelagophyceae	*Aureococcus anophagefferens*	1 vs m
ASV3052	Ochrophyta	Pelagophyceae	*Aureococcus anophagefferens*	1 vs m
ASV784	Bacillariophyta	Bacillariophyceae	Cylindrotheca closterium*	1 vs m
ASV1028	Bacillariophyta	Bacillariophyceae	*Cylindrotheca closterium*	1 vs m
ASV3121	Bacillariophyta	Bacillariophyceae	*Cylindrotheca closterium*	1 vs m
ASV844	Dinoflagellata	Dinophyceae	Dinophysis acuminata*	1 vs m
ASV951	Dinoflagellata	Dinophyceae	*Dinophysis acuminata*	1 vs m
ASV1117	Dinoflagellata	Dinophyceae	*Dinophysis acuminata*	1 vs m
ASV1123	Dinoflagellata	Dinophyceae	*Dinophysis acuminata*	1 vs m
ASV1202	Dinoflagellata	Dinophyceae	*Dinophysis acuminata*	1 vs m
ASV1776	Dinoflagellata	Dinophyceae	*Dinophysis acuminata*	1 vs m
ASV1827	Dinoflagellata	Dinophyceae	*Dinophysis acuminata*	1 vs m
ASV1945	Dinoflagellata	Dinophyceae	*Dinophysis acuminata*	1 vs m
ASV862	Haptophyta	Coccolithophyceae	Emiliania huxleyi*	1 vs m
ASV1340	Haptophyta	Coccolithophyceae	*Emiliania huxleyi*	1 vs m
ASV2827	Haptophyta	Coccolithophyceae	*Emiliania huxleyi*	1 vs m
ASV3214	Haptophyta	Coccolithophyceae	*Emiliania huxleyi*	1 vs m
ASV1195	Bacillariophyta	Coscinodiscophyceae	Guinardia striata*	1 vs m
ASV1356	Bacillariophyta	Coscinodiscophyceae	*Guinardia striata*	1 vs m
ASV1599	Bacillariophyta	Coscinodiscophyceae	*Guinardia striata*	1 vs m
ASV1403	Dinoflagellata	Dinophyceae	Gymnodinium aureolum**	1 vs m
ASV2880	Dinoflagellata	Dinophyceae	*Gymnodinium aureolum*	1 vs m
ASV2977	Dinoflagellata	Dinophyceae	*Gymnodinium aureolum*	1 vs m
ASV3144	Dinoflagellata	Dinophyceae	*Gymnodinium aureolum*	1 vs m
ASV3167	Dinoflagellata	Dinophyceae	*Gymnodinium aureolum*	1 vs m
ASV15	Dinoflagellata	Dinophyceae	Heterocapsa rotundata**	1 vs m
ASV40	Dinoflagellata	Dinophyceae	*Heterocapsa rotundata*	1 vs m
ASV118	Dinoflagellata	Dinophyceae	*Heterocapsa rotundata*	1 vs m
ASV630	Dinoflagellata	Dinophyceae	*Heterocapsa rotundata*	1 vs m
ASV42	Dinoflagellata	Dinophyceae	Karlodinium veneficum **	1 vs m
ASV147	Dinoflagellata	Dinophyceae	Karlodinium veneficum	1 vs m
ASV569	Haptophyta	Prymnesiophyceae	Phaeocystis globosa*	1 vs m
ASV777	Haptophyta	Prymnesiophyceae	*Phaeocystis globosa*	1 vs m
ASV790	Dinoflagellata	Dinophyceae	Phalacroma mitra*	1 vs m
ASV793	Dinoflagellata	Dinophyceae	*Phalacroma mitra*	1 vs m
ASV798	Dinoflagellata	Dinophyceae	*Phalacroma mitra*	1 vs m
ASV864	Dinoflagellata	Dinophyceae	*Phalacroma mitra*	1 vs m
ASV871	Dinoflagellata	Dinophyceae	*Phalacroma mitra*	1 vs m
ASV881	Dinoflagellata	Dinophyceae	*Phalacroma mitra*	1 vs m
ASV889	Dinoflagellata	Dinophyceae	*Phalacroma mitra*	1 vs m
ASV925	Dinoflagellata	Dinophyceae	*Phalacroma mitra*	1 vs m
ASV930	Dinoflagellata	Dinophyceae	*Phalacroma mitra*	1 vs m
ASV937	Dinoflagellata	Dinophyceae	*Phalacroma mitra*	1 vs m
ASV972	Dinoflagellata	Dinophyceae	*Phalacroma mitra*	1 vs m
ASV986	Dinoflagellata	Dinophyceae	*Phalacroma mitra*	1 vs m
ASV1009	Dinoflagellata	Dinophyceae	*Phalacroma mitra*	1 vs m
ASV1041	Dinoflagellata	Dinophyceae	*Phalacroma mitra*	1 vs m
ASV1062	Dinoflagellata	Dinophyceae	*Phalacroma mitra*	1 vs m
ASV1068	Dinoflagellata	Dinophyceae	*Phalacroma mitra*	1 vs m
ASV1079	Dinoflagellata	Dinophyceae	*Phalacroma mitra*	1 vs m
ASV1087	Dinoflagellata	Dinophyceae	*Phalacroma mitra*	1 vs m
ASV1098	Dinoflagellata	Dinophyceae	*Phalacroma mitra*	1 vs m
ASV1099	Dinoflagellata	Dinophyceae	*Phalacroma mitra*	1 vs m
ASV1127	Dinoflagellata	Dinophyceae	*Phalacroma mitra*	1 vs m
ASV1129	Dinoflagellata	Dinophyceae	*Phalacroma mitra*	1 vs m
ASV1148	Dinoflagellata	Dinophyceae	*Phalacroma mitra*	1 vs m
ASV1159	Dinoflagellata	Dinophyceae	*Phalacroma mitra*	1 vs m
ASV1161	Dinoflagellata	Dinophyceae	*Phalacroma mitra*	1 vs m
ASV1192	Dinoflagellata	Dinophyceae	*Phalacroma mitra*	1 vs m
ASV1199	Dinoflagellata	Dinophyceae	*Phalacroma mitra*	1 vs m
ASV1206	Dinoflagellata	Dinophyceae	*Phalacroma mitra*	1 vs m
ASV1239	Dinoflagellata	Dinophyceae	*Phalacroma mitra*	1 vs m
ASV1266	Dinoflagellata	Dinophyceae	*Phalacroma mitra*	1 vs m
ASV1285	Dinoflagellata	Dinophyceae	*Phalacroma mitra*	1 vs m
ASV1327	Dinoflagellata	Dinophyceae	*Phalacroma mitra*	1 vs m
ASV1417	Dinoflagellata	Dinophyceae	*Phalacroma mitra*	1 vs m
ASV1432	Dinoflagellata	Dinophyceae	*Phalacroma mitra*	1 vs m
ASV1493	Dinoflagellata	Dinophyceae	*Phalacroma mitra*	1 vs m
ASV1499	Dinoflagellata	Dinophyceae	*Phalacroma mitra*	1 vs m
ASV1517	Dinoflagellata	Dinophyceae	*Phalacroma mitra*	1 vs m
ASV1537	Dinoflagellata	Dinophyceae	*Phalacroma mitra*	1 vs m
ASV1558	Dinoflagellata	Dinophyceae	*Phalacroma mitra*	1 vs m
ASV1604	Dinoflagellata	Dinophyceae	*Phalacroma mitra*	1 vs m
ASV1630	Dinoflagellata	Dinophyceae	*Phalacroma mitra*	1 vs m
ASV1633	Dinoflagellata	Dinophyceae	*Phalacroma mitra*	1 vs m
ASV1663	Dinoflagellata	Dinophyceae	*Phalacroma mitra*	1 vs m
ASV1682	Dinoflagellata	Dinophyceae	*Phalacroma mitra*	1 vs m
ASV1750	Dinoflagellata	Dinophyceae	*Phalacroma mitra*	1 vs m
ASV1777	Dinoflagellata	Dinophyceae	*Phalacroma mitra*	1 vs m
ASV1834	Dinoflagellata	Dinophyceae	*Phalacroma mitra*	1 vs m
ASV2040	Dinoflagellata	Dinophyceae	*Phalacroma mitra*	1 vs m
ASV2462	Dinoflagellata	Dinophyceae	*Phalacroma mitra*	1 vs m
ASV2663	Dinoflagellata	Dinophyceae	*Phalacroma mitra*	1 vs m
ASV2667	Dinoflagellata	Dinophyceae	*Phalacroma mitra*	1 vs m
ASV2943	Dinoflagellata	Dinophyceae	*Phalacroma mitra*	1 vs m
ASV3168	Dinoflagellata	Dinophyceae	*Phalacroma mitra*	1 vs m
ASV1093	Dinoflagellata	Dinophyceae	*Phalacroma mitra*	1 vs m
ASV2324	Haptophyta	Coccolithophyceae	Prymnesium polylepis**	1 vs m
ASV2765	Haptophyta	Coccolithophyceae	*Prymnesium polylepis*	1 vs m
ASV2961	Haptophyta	Coccolithophyceae	*Prymnesium polylepis*	1 vs m
ASV2969	Haptophyta	Coccolithophyceae	*Prymnesium polylepis*	1 vs m
ASV934	Bacillariophyta	Bacillariophyceae	Pseudo-nitzschia seriata*	1 vs m
ASV952	Bacillariophyta	Bacillariophyceae	*Pseudo-nitzschia seriata*	1 vs m
ASV957	Bacillariophyta	Bacillariophyceae	*Pseudo-nitzschia seriata*	1 vs m
ASV960	Bacillariophyta	Bacillariophyceae	*Pseudo-nitzschia seriata*	1 vs m
ASV969	Cyanobacteria	Cyanophyceae	Trichodesmium erythraeum *	1 vs m
ASV1218	Cyanobacteria	Cyanophyceae	Trichodesmium erythraeum	1 vs m
ASV1842	Cyanobacteria	Cyanophyceae	Trichodesmium erythraeum	1 vs m
ASV2299	Cyanobacteria	Cyanophyceae	Trichodesmium erythraeum	1 vs m
ASV2619	Cyanobacteria	Cyanophyceae	Trichodesmium erythraeum	1 vs m
ASV2769	Cyanobacteria	Cyanophyceae	Trichodesmium erythraeum	1 vs m
ASV2953	Cyanobacteria	Cyanophyceae	Trichodesmium erythraeum	1 vs m

a 1 vs 1 indicates one ASV-one species relationship; 1 vs m indicates one species-multiple ASVs relationship. Relevant information of HAB species can be found in references 1, 65, 66.

b The symbol * is for HAB species that have been reported in the SCS.

c The symbol ** is for HAB species that have not been reported in the SCS.

Of the 19 annotated HAB species, 8, 5, 3, 1, 1, and 1 species belonged to Dinoflagellata, Bacillariophyta, Haptophyta, Ochrophyta, Chlorophyta, and Cyanobacteria, respectively. Among the eight dinoflagellates, all species were from the class Dinophyceae. In addition, we found that different HAB species usually have different distribution patterns in the sampling sites (Fig. S4). Furthermore, different ASVs of the same HAB species displayed differential geographical distribution patterns, such as those ASVs annotated as Phalacroma mitra (54 ASVs) and *A. anophagefferens* (27 ASVs). In particular, HAB species *A. anophagefferens* and Phaeocystis globosa were more widely distributed in the seamount region of Xianbei (XB) than in the Dongsha (DS) and Xisha (XS) sea areas. In contrast, *P. mitra* species was widely distributed in three sea areas.

**FIG 6:**
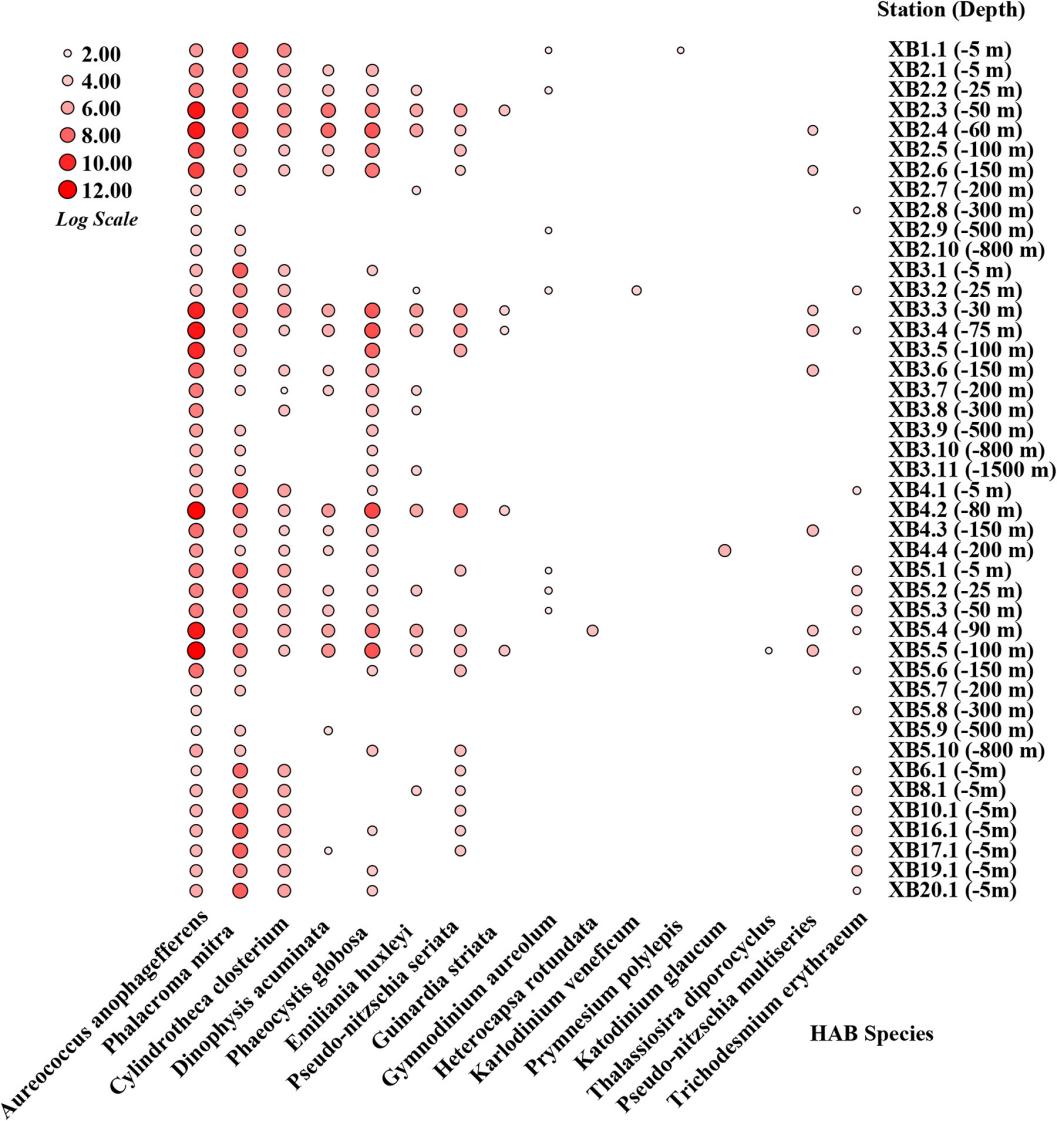
Distribution and abundance of 16 HAB species in the seamount region of Xianbei along the depth gradients. The size of the red circle represents the percentage of the relative abundance.

To reveal the differences in HAB species composition and distribution along the depth gradients in the seamount region of Xianbei, we further analyzed data from 19 HAB species except for 3 HAB species Gonyaulax polygramma, Alexandrium leei, and *P. parkeae* (Fig. 6). The results showed that several HAB species followed clear depth-specific trends in XB. The *A. anophagefferens* species usually dominated the shallow waters (−5, −25, and −50 m) and deep chlorophyll maximum (DCM) (−60 m), but its relative abundance decreased with increasing depth, especially at sites XB2 and XB3. Furthermore, *A. anophagefferens* was the only HAB species distributed at all sites in XB, both in deep and shallow waters. The relative contribution of *P. mitra* showed a similar trend, with relative abundance generally increasing with the increase in depth, at sites XB2, XB3, XB4, and XB5. Although *P. globosa* was not present at all sites, it showed a significant downward trend as water depth increases, especially at the XB3 site, ranging from −30 m to −1,500 m. The contribution of the other HAB species was small across all water depths but generally higher in shallow than deep waters.

### Phylogenetic network analyses of HAB species.

**FIG 7:**
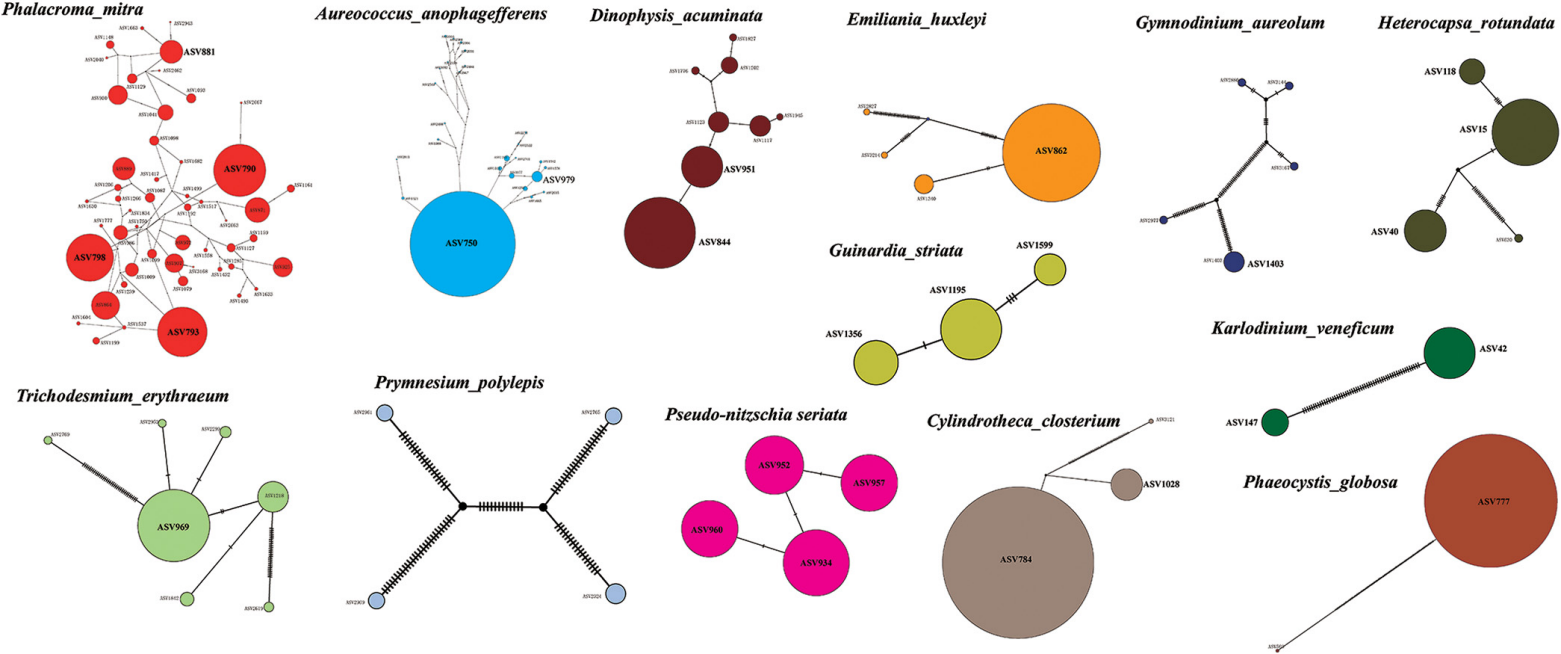
Phylogenetic network analyses of dominant HAB species identified in the seamount region of Xianbei. The size of the circles represents the relative abundance of each ASV.

Phylogenetic haplotype networks were constructed using an agglomerative approach where clusters were progressively combined with one or more connecting edges (Fig. 7). Based on the analysis results, we found that the main branches connecting the nodes showed little reticulation in HAB species, including *D. acuminata*, *E. huxleyi*, *G. striata*, *G. aureolum*, *H. rotundata*, K. veneficum, *C. closterium*, *P. globosa*, *P. polylepis*, Pseudo-nitzschia seriata, and *T. erythraeum*. However, the network structures were rather complex in HAB species *P. mitra* (Fig. 7, orange) and *A. anophagefferens* (Fig. 7, blue), suggesting the possibility of gene flow among different haplotypes.

Among the 13 dominant HAB species, phylogenetic network analysis of the 54 ASVs annotated as *P. mitra* displayed the highest diversity. Furthermore, the haplotype network also showed ASV790, ASV793, and ASV798 were the three haplotypes with the highest proportion. Notably, among the 27 ASVs identified in the species *A. anophagefferens*, ASV750 was the most abundant haplotype. For each HAB species identified in the seamount region of Xianbei, a high level of intrapopulation genetic diversity was generally uncovered, indicating the existence of a large number of cryptic species that have not been characterized previously.

### Correlation of HAB species with environmental factors in seamount region of Xianbei.

The water temperature (°C), dissolved oxygen (mg/L), density (kg/m^3^), nitrogen saturation (mg/L) were 2.77 to 30.31°C, 2.82 to 7.05 mg/L, 1,013.35 to 1,034.57 kg/m^3^, and 10.97 to 16.68 mg/L, respectively (Table S3 and Fig. S5). Notably, these major environmental factors, including water temperature and oxygen, had an obvious downward trend from shallow to deep waters. In contrast to the first two factors, the values of density and nitrogen saturation generally increased with increasing water depth.

To explore the impact of environmental factors on HAB species, the correlation between each HAB species and environmental factors was calculated. Through RDA analysis, we found that HAB species at different sites were affected by different environmental factors in the seamount region of Xianbei (Fig. S6). The results of RDA showed that the two axes of the RDA plot explained 94.45% and 5.48% of the cumulative variances in the species-environment relationship, respectively. While the two axes together explained 99.93% of the variation. Dissolved oxygen and temperature were positively correlated with these assemblages, which were two significant environmental factors affecting the community of HAB species, compared to other factors. The biomass of *A. anophagefferens* and *P. mitra* were positively correlated with oxygen and temperature and negatively correlated with density and nitrogen saturation in the water of the seamount region of Xianbei.

### Similarity between coastal waters and the seamount region of Xianbei of HAB species.

**FIG 8:**
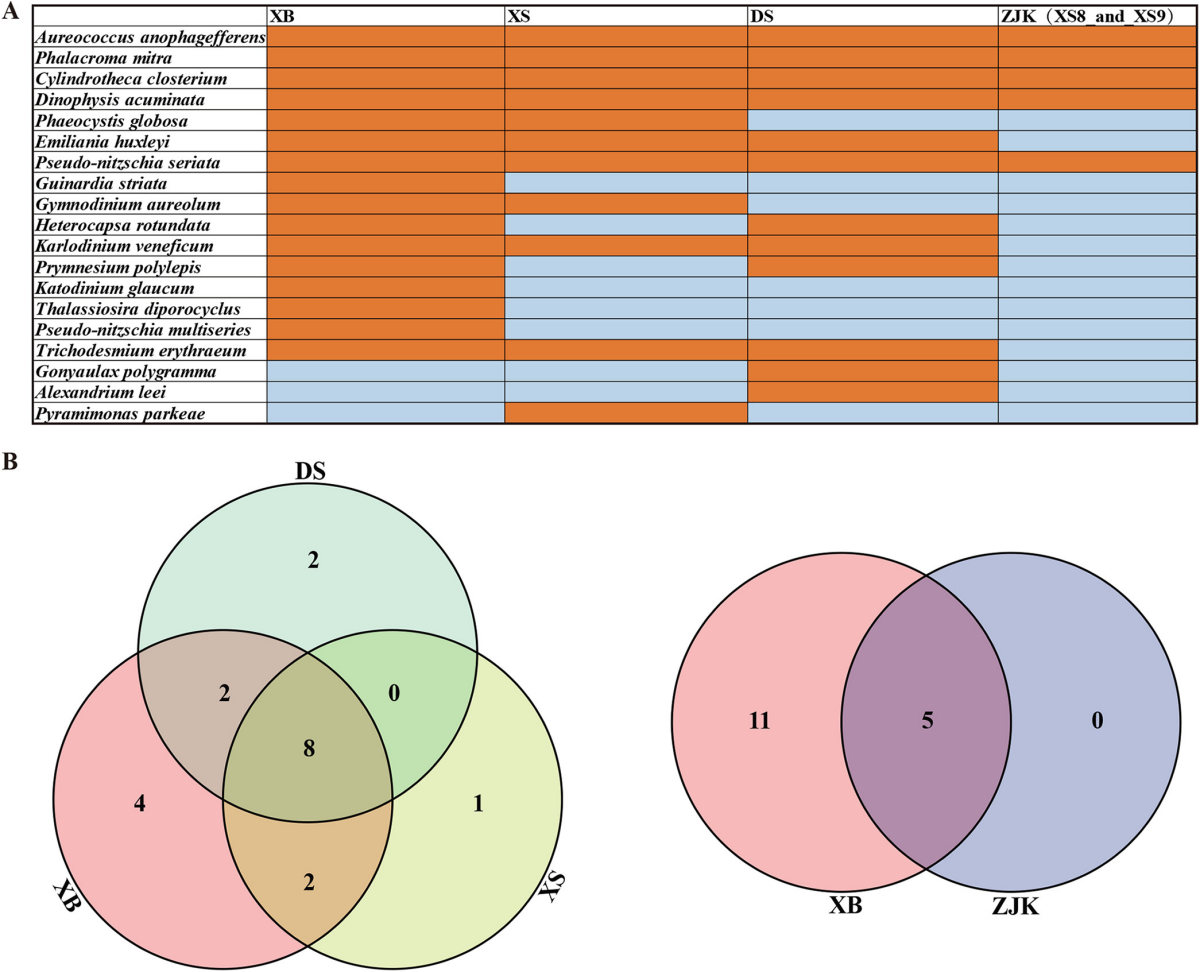
Comparison of HAB species in the seamount region of Xianbei (XB), Dongsha (DS), Xisha (XS), and the Pearl River Estuary (ZJK). (A) The presence (orange) and absence (blue) pattern of 19 HAB species in XB, DS, XS, and ZJK. (B) Venn diagrams of HAB species in XB, DS, and XS and in XB and ZJK.

In this study, the results displayed 19 annotated HAB species at all sampling sites in XB, XS, DS, and ZJK (XS8 and XS9) (Fig. 8A). To reflect the differences and similarities in HAB species composition more intuitively, a Venn diagram and pie plot were constructed at the species level in this study (Fig. 8B). The number of HAB species at the sampling sites in XB, XS, DS and ZJK was 16, 11, 12, and 5, respectively. Among these sampling sites, we found 13 shared HAB species, including 8 species in Dinoflagellata and 5 species in Bacillariophyta. Compared with the other areas, XB had a characteristic community of HAB species. The number of shared HAB species in the three sampling regions including XB, XS, and DS reached 8, with a proportion of 42.11%. Furthermore, the number of shared HAB species between XB and ZJK was five, with a proportion of 45.45%, including *A. anophagefferens* (Ochrophyta), *P. mitra* (Dinoflagellata), *C. Closterium* (Bacillariophyta), *D. acuminata* (Dinoflagellata), and *Pseudo-nitzschia seriata* (Bacillariophyta).

## DISCUSSION

Seamounts are “the least understood habitats on earth” and tend to have high biomass and biodiversity, which are not only an important area of marine fishery in the world but also hot areas of ecological environment research (18). However, compared to other ocean areas, little research has been reported on phytoplankton in seamounts of the SCS, especially on HAB species.

Several universal molecular markers with their versatility have been used to analyze the species diversity and community structure of microbial populations (22). Generally, lots of studies characterized the phytoplankton community and diversity with the short hypervariable regions (V1 to V9) of the 18S rDNA gene, but Cyanobacteria taxa could not be detected simultaneously (34). Fortunately, the metabarcoding approach with the universal primer set (515Y/926R) is ideal for quantifying marine primary producers, including both eukaryotic algae and cyanobacteria (25, 35). Therefore, the primer set (515Y/926R) was chosen as the best-performing primer pair for amplicon sequencing in this study. Nevertheless, there were some inevitable shortcomings to evaluate the abundance solely relying on high-throughput sequencing, mainly because the relative abundance of some species might be overestimated (36). First of all, water samples usually consist of nucleic acids derived from living, dormant, and inactive/dead phytoplankton cells, as well as extracellular free DNA molecules. Second, rDNA gene sequences of some dinoflagellate genomes have a large number of repeats, making it difficult to directly link read numbers to the abundance of an individual organism (28, 37, 38). Third, PCR amplification bias can result in a large amount of missing data or an overestimation of diversity and may lead to a misinterpretation of species composition, including the dominance of certain groups (39, 40). Therefore, although the environmental water samples were not identified in this study based on morphology, we need to apply morphological identification to quantify phytoplankton species cells more accurately in the future.

In this study, 9 divisions and 22 classes of phytoplankton were detected by the metabarcoding approach. Among them, Cyanobacteria was found to be the dominant division, exhibiting the highest richness and relative abundance in samples of the SCS. On the one hand, the SCS was characterized as an oligotrophic environment, where cyanobacteria could adapt well and grow better than eukaryotic algae (41). Compared with other databases including PR^2^ and DINOREF (42, 43), the rRNA database SILVA 138 had a richer and more complete classification (44). However, in this study, a relatively higher abundance of unclassified ASVs (NA cyanobacteria) appeared in Xianbei sites. These unclassified ASVs may be new species, but it is also possible that a large number of unmatched sequences are caused by the inadequacy of the reference database (45, 46). In addition, Dinoflagellata was the most dominant group, followed by Chlorophyta in the eukaryotic algae survey. Dinoflagellata was also previously reported to be the most dominant division in the Western Pacific regions, which may due to environmental conditions (7, 47). However, among 2,776 phytoplankton ASVs, only a small portion (939 ASVs, 33.83%) could be annotated as single species, whereas the vast majority (1,837, 66.17%) could not be annotated, suggesting that phytoplankton (especially HAB species) in the SCS are seriously underestimated. Because of the limitation of current phytoplankton databases, most ASV sequences could not be properly annotated in this study. Thus, a more comprehensive phytoplankton reference database is urgently needed.

Additionally, in this study, the results of diversity analysis showed fluctuations in marine phytoplankton diversity (including eukaryotic algae and cyanobacteria) at different sample sites in the SCS. The diversity indexes of samples XB16.1 and XB4.1 in the XB sampling site were higher than others, suggesting a stable phytoplankton community. Our results also showed that phytoplankton diversity and richness were higher in XB than in DS and XS off the seamount regions. This phenomenon was also observed in other seamounts in previous studies. For example, Dai et al. (18) showed that phytoplankton biomass was enhanced on the seamount in the tropical western Pacific. Meanwhile, their results of physicochemical characteristics supported the classic hypothesis of the seamount effect. In the classic hypothesis, upwelling in the euphotic zone is an important process contributing to high biomass above seamounts, because it can advance vertical mixing and supply nutrients to the upper zone to promote marine primary productivity (15). Moreover, previous studies also found increased phytoplankton biomass near the shallow seamounts in the northeastern Atlantic, including the Great Meteor Tablemount (GMT) (48), Seine seamount (49), and Gorringe Bank (19). However, some studies did not find this phenomenon that phytoplankton biomass was enhanced on the seamount (50). Even on the same seamount, the phenomenon did not exist at all times (51). In addition, in this study the diversity of phytoplankton community with a slight downward trend from the open sea to the Pearl River Estuary, which suggested that the environmental conditions in the open sea were less stressed near the Pearl River Estuary. As global warming and eutrophication due to the increased nutrient loading from human activities, HABs occurred frequently in the coastal waters near the Pearl River Estuary in recent decades (14, 52). This may be the reason for the low diversity of phytoplankton species in nearshore waters.

Several studies on the diversity of microbial eukaryotes in the different depths of water have been carried out in the SCS (38, 53). In the present study, the analysis of alpha diversity along the depth gradients in the seamount region of Xianbei suggested that the communities of eukaryotic algae and cyanobacteria were both shaped by water depth. In the both eukaryotic algae and cyanobacteria surveys, the richness index in the DCM (−60, −75, −80, and −90 m, XB_DCM) was relatively higher than that of the samples from the shallow (−5, −25, and −50 m, XB_shallow), middle (−100, −150, and −200 m, XB_middle) and deep water (−300, −500, −800, and −1,500 m, XB_deep). Dai et al. (18) also showed consistent results that picophytoplankton was the dominant group in the DCM. However, the diversity of the XB_shallow group was the highest in the eukaryotic algae survey and lowest in the cyanobacteria survey in the different four groups. Light is the driving force of photosynthesis, but excessive light could inhibit photosynthesis and damage phytoplankton growth (41). In summer, the surface phytoplankton community in the SCS basin area is dominated by picocyanobacteria, *Prochlorococcus*, and *Synechococcus* (54). Moreover, lots of eukaryotic algae belong to mixotrophy (e.g., Karlodinium veneficum), which also can survive by grazing on cryptophytes, diatoms, dinoflagellates and prokaryotes (55, 56). Combined with the above analysis, we found the major groups of Dinoflagellate, Ochrophyta, and Bacillariophyta in the depth range from −200 to −1,000 m. Similarly, a high richness of Cyanobacteria (mainly *Prochlorococcus* and *Synechococcus*) was found in the deep sea. Previous studies have reported the presence of phytoplankton cells and healthy photoautotrophic cells, especially diatoms, in the deep sea (down to −4,000 m) (57). In addition, Xu et al. (38) have confirmed the presence of not only live but also active photoautotrophic microbial eukaryotes in the deep sea in the SCS.

Our analyses revealed numerous HAB species, many of which were first reported in the SCS, demonstrating the advantage of the metabarcoding approach in detecting HAB species composition in the phytoplankton community. Among the ASVs, 133 ASVs were annotated to 19 HAB species with one ASV-one species relationship and multiple ASV-one species relationships. Of these 19 annotated HAB species, 6 were first reported in the SCS, while 16 (84.21%) were found in XB. In this study, we revealed that the HAB species in XB significantly more than the surrounding sea areas in the SCS. Among the 16 HAB species, *A. anophagefferens*, *P. mitra*, and *P. globosa* were more widely distributed in XB. For instance, the spherical, nonmotile, picoplanktonic (2 to 3 μm) pelagophyte *A. anophagefferens* had caused “brown tides” and consequently disastrous mortalities in the cultured shellfish and disruptive effects on seagrass beds and other natural resources in the United States, South Africa, and China (11). However, *A. anophagefferens* had been greatly overlooked in phytoplankton studies for a long time. Its small size and morphological similarity to other algal species make it extremely difficult to identify. In this study, 27 different ASVs were assigned to *A. anophagefferens*, implying that it had a diverse genetic diversity and was not only mainly distributed in XB but also inhabited the deep sea (down to −1,500 m). Early studies showed that different genetic diversity of an algal species may have different physiological characteristics and distribution patterns, and may make different contributions to the formation of algal blooms (9, 58, 59). Furthermore, seamounts not only provided special habitats for species reproduction but also provided abundant seeds for coastal regions as a “seed bank” (15). Previous studies have shown that physical processes will form a “seamount effect,” which promotes phytoplankton growth and increases primary and secondary productivity. Therefore, the relatively high abundance of *A. anophagefferens* may be influenced by the seamount. However, it cannot be ruled out that the relatively high abundance of *A. anophagefferens* is caused by other factors, such as sampling and sequencing method, season, water depth, water current, etc. Although there were no records of blooms caused by *A. anophagefferens* in the SCS, measures should be taken to prevent such an occurrence, rather than mitigating the effects when “brown tides” occur.

There were 5 shared HAB species in all sampling sites. Among them, *A. anophagefferens* and *P. mitra* showed higher abundance in XB, which were frequently found in marine water, especially in coastal waters, and often became the dominant species of HABs. As reported previously, *P. globosa* was one of the most frequent HAB-forming species along the coast of Guangdong and displayed a rich genetic diversity in the field (14, 58, 59). The cooccurrence of 5 HAB species in the Xianbei seamount region of SCS, and in the ZJK suggested a potential correlation among these distinct ocean regions, possibly via ocean currents. Dai et al. (60) confirmed their hypothesis that the blooms of Prorocentrum donghaiense developed from the population at the Taiwan Warm Current (TWC) front in the East China Sea (ECS), suggesting the role of the ocean current front as a “pelagic seed bank” to dinoflagellate blooms. In agreement with the results obtained in this study, previous findings demonstrated that ocean currents can carry HAB species into coastal waters (7, 60). For future studies, seasonal water samples of other seamount regions of the SCS should be collected to do a comparative study.

### Conclusions.

In this study, we described the phytoplankton diversity, community composition, and spatial distribution of HAB species in the SCS during August and September of 2021. Picophytoplankton, mainly *Prochlorococcus* and *Synechococcus*, were the dominant groups in the surface waters of the studied areas. Meanwhile, our results also showed that phytoplankton diversity and richness were higher in the seamount region of Xianbei than in the neighboring open ocean, consistent to the classic “seamount effect” hypothesis. To the best of our knowledge, this was the first study of focusing on the diversity and distribution of HAB species in the seamount of Xianbei in the SCS. Moreover, our study highlighted the strength of the ASV-based metabarcoding approach in discovering undetected or unreported HAB algal species in previous studies. Our results also revealed the presence of picophytoplankton in the dark and deep SCS. In addition, the results of the similarity analysis of HAB species composition between XB and ZJK (Pearl River Estuary) indicated that there might be a tight connection between seamount and coastal waters. In the future, further efforts encompassing morphological studies as well as environmental surveys combined with the metabarcoding approach in various seamounts will surely reveal more “hidden” diversity and composition of poorly understood HAB species.

## MATERIALS AND METHODS

### Location and sample collection.

The sampling sites are located in the South China Sea (16°10′-22°02′ N, 114°22′-118°21′ E), which is one of the largest marginal seas in the world with an average depth of 1,212 kilometers and is a semiclosed sea surrounded by nine countries (Fig. 1). The expedition, which was carried out during August and September in 2021 on research vehicle “Shenkuo,” was supported by the Southern Marine Science and Engineering Guangdong Laboratory (Zhuhai). Water samples were collected at 30 sampling sites belonging to three ocean regions in the SCS. Among these sites, XS3.1 to XS9.1 are located in the Xisha (XS) sea areas, particularly, XS8.1 and XS9.1 are located near the mouth of the Pearl River Estuary. In addition, DS6.1 to DS17.1 are located in the Dongsha (DS) sea areas. XB1.1 to XB20.1 are located in the seamount region of Xianbei (XB). Among these sites, the numbers after the decimal point represent different depths.

At each sampling site, 2 L seawater at different depths was collected, with the sampling depths ranging from −5 m (i.e., surface) to −1,500 m, using a rosette sampler equipped with a SeaBird CTD (conductivity-temperature-depth) system (Ocean Test Equipment, Inc., Fort Lauderdale, FL, USA). Water samples were first prefiltered using 200 μm mesh to remove large suspended solids, larger zooplankton and phytoplankton, followed by a secondary filtration through a 0.2 μm polycarbonate membranes (Millipore, USA) using a vacuum filtration pump with negative pressure below 50 kPa. The filter membranes were transferred in tubes and were then snap-frozen in liquid nitrogen and stored at −80°C until DNA extraction. The water temperature (°C), dissolved oxygen (mg/L), density (kg/m^3^), and nitrogen saturation (mg/L) were measured for all samples collected during the expedition. Major physicochemical characteristics were measured using a SeaBird CTD system (Ocean Test Equipment, Inc. Fort Lauderdale, FL, USA) on board.

### DNA extraction, PCR amplification, and high-throughput sequencing.

Extraction and purification of environmental DNA from the samples’ membranes were carried out using the HP Plant DNA kit (Omega, USA) according to a modified manufacturer's protocol previously described by Huang et al. (27). Finally, DNA concentrations were quantified using a Nanodrop 2000 instrument (Thermo Fisher Scientific, USA). The extracted genomic DNA samples were stored at −80°C until being used as the temple for PCR amplification. DNA was amplified with the 515Y/926R primers (515Y: 5′-GTGYCAGCMGCCGCGGTAA-3′/926R: 5′-CCGYCAATTYMTTTRAGTTT-3′) (35) using 0.5 ng of DNA template in a 25-mL reaction mixture with a final primer concentration of 0.3 mM. Both forward and reverse primers were tagged with Illumina adapter and sample-specific barcodes. All primers were synthesized by Sangon Biotech Co., Ltd. (Shanghai, China). Thermal cycling consisted of initial denaturation at 95°C for 120 s, followed by 30 cycles of denaturation at 95°C for 45 s, annealing at 50°C for 45 s, elongation at 68°C for 90 s, and a final extension at 68°C for 300 s.

The degradation and contamination of PCR products were examined on 2% agarose gels, followed by purifying with the Qiagen Gel Extraction kit (Qiagen, Germany) according to the manufacturer’s instructions. The libraries were generated using a TruSeq DNA PCR-Free Sample Preparation kit (Illumina, USA) following the manufacturer’s recommendations. The quality of sequencing libraries was assessed on the Qubit 2.0 Fluorometer (Thermo Scientific) and Agilent Bioanalyzer 2100. The libraries were subsequently sequenced on an Illumina NovaSeq platform (Illumina, Santa Clara, CA, USA; Novogene, Beijing, China), and 250-bp paired-end reads were generated.

### Data processing of amplicon sequencing data.

Amplicon sequences were analyzed using the QIIME2 toolkit v2021.11 (61) following the Fuhrman lab protocol (https://github.com/jcmcnch/eASV-pipeline-for-515Y-926R) as previously described (25, 62). Briefly, raw Illumina NovaSeq sequencing reads were first trimmed using cutadapt v3.5 to remove sequencing adaptors and PCR primers with an error rate of 0.2. Then clean reads were further split into 16S and 18S rRNA pools using a custom 16S/18S databases derived from SILVA 138 rRNA database (from Latin silva, forest, http://www.arb-silva.de/) (44) and Protist Ribosomal Reference database (https://github.com/pr2database/pr2database, PR^2^) (42). For each pool, amplicon sequence variants (ASVs) were constructed separately using the DADA2 (29) R package on the QIIME2 platform. Since the forward and reverse shotgun reads do not overlap for 18S rRNA amplicon data, we trimmed the forward reads to 210 bp and reverse reads to 170 bp following the protocol’s instructions using BBduk.sh and directly concatenated the trimmed forward and reverse reads using fuse.sh from the BBtools suite (https://sourceforge.net/projects/bbmap/). Then the concatenated 18S rRNA reads were denoised using the DADA2 denoise-single command with default parameters. After that, 16S rRNA ASVs were taxonomically classified against the SILVA 138 database, and Chloroplast 16S rRNA ASVs and 18S rRNA ASVs were classified against the PR^2^ database. ASVs assigned to the mitochondria family were discarded in the following analysis.

### Bioinformatics analysis.

Only ASVs that were supported by two or more reads in at least one sample were included in further analysis. The classification of phytoplankton ASVs in this study was based on algaebase (http://www.algaebase.org). For ASVs that were not successfully annotated (including the ASVs identified as chloroplasts) were further annotated by searching against NCBI NT database using BLASTN (E value = 10^−6^) with a percent identity threshold of 99%. Finally, ASVs were divided then into two groups, depending on whether a one-to-one or many-to-one relationship can be established between ASVs and phytoplankton species.

The rarefaction curves were plotted with ASVs richness for each sample using the vegan package in R (https://www.r-project.org). Other community diversity parameters were also analyzed using vegan, including Richness (ASV richness), Chao1, Shannon diversity, Simpson diversity, Pielou’s evenness, ACE, and Good’s coverage (27). The sampling locations were visualized using a grid-based mapping program Surfer 16.0 (Golden Software). In addition to analyzing the relative abundance of phytoplankton at the species level, we also analyzed the richness indexes and relative abundance of these samples at the division and the class level using R. The phylogenetic haplotype networks were constructed using the statistical parsimony algorithm implemented in the TCS network (63). Haplotype networks were visualized in PopART v1.7 (64). Correlations between the community of HAB species and water quality parameters were identified by linear model-based redundancy analysis (RDA) in R using Spearman's correlation coefficients.

### Data availability statement.

The data sets generated during the current study are available from the corresponding authors on request. Illumina raw sequences are available on NCBI’s sequence read archive (SRA) at BioProject: PRJNA880762.

## References

[1] Gu H, Wu Y, Lu S, Lu D, Tang Y, Qi Y. 2022. Emerging harmful algal bloom species over the last four decades in China. Harmful Algae 111:102059. doi:10.1016/j.hal.2021.102059. 35016757

[2] Xiao X, Agusti S, Pan Y, Yu Y, Li K, Wu J, Duarte CM. 2019. Warming amplifies the frequency of harmful algal blooms with eutrophication in Chinese coastal waters. Environ Sci Technol 53:13031–13041. doi:10.1021/acs.est.9b03726. 31609108

[3] Yu RC, Lü SH, Qi YZ, Zhou MJ. 2020. Progress and perspectives of harmful algal bloom studies in China. Oceanol Limnol Sin 51:769–788.

[4] Hu ZX, Shang LX, Deng YY, Tang YZ. 2020. Retrospect and prospect: studies on geographical expansion of resting cysts of non-indigenous harmful algal bloom (HAB)-forming dinoflagellates via ships’ ballast tanks. Mar Sci 44:103–115.

[5] Yu ZM, Chen NS. 2019. Emerging trends in red tide and major research progresses. Oceanol Limnol Sin 50:474–486.

[6] Glibert PM, Berdalet E, Burford MA, Pitcher GC, Zhou M. 2018. Global ecology and oceanography of harmful algal blooms. Cham, Switzerland, Springer Nature.

[7] Xu Q, Wang CZ, Xu KD, Chen NS. 2021. Metabarcoding analysis of harmful algal bloom species in the Western Pacific Seamount regions. Int J Environ Res Public Health 18:11470. doi:10.3390/ijerph182111470. 34769984 PMC8582749

[8] Doblin MA, Popels LC, Coyne KJ, Hutchins DA, Cary SC, Dobbs FC. 2004. Transport of the harmful bloom alga *Aureococcus anophagefferens* by oceangoing ships and coastal boats. Appl Environ Microbiol 70:6495–6500. doi:10.1128/AEM.70.11.6495-6500.2004. 15528511 PMC525227

[9] Gobler CJ, Sunda WG. 2012. Ecosystem disruptive algal blooms of the brown tide species, *Aureococcus anophagefferens* and *Aureoumbra lagunensis*. Harmful Algae 14:36–45. doi:10.1016/j.hal.2011.10.013.

[10] Zhang QC, Qiu LM, Yu RC, Kong FZ, Wang YF, Yan T, Gobler CJ, Zhou MJ. 2012. Emergence of brown tides caused by *Aureococcus anophagefferens* Hargraves et Sieburth in China. Harmful Algae 19:117–124. doi:10.1016/j.hal.2012.06.007.

[11] Tang YZ, Ma Z, Hu Z, Deng Y, Yang A, Lin S, Yi L, Chai Z, Gobler CJ. 2019. 3,000 km and 1,500-year presence of *Aureococcus anophagefferens* reveals indigenous origin of brown tides in China. Mol Ecol 28:4065–4076. doi:10.1111/mec.15196. 31468654

[12] Song L, Wu J, Du J, Li N, Song G, Wang K, Sun M, Wang P. 2019. The characteristics and distribution of eukaryotic phytoplankton community in Liaodong Bay, China. Ocean Sci J 54:183–203. doi:10.1007/s12601-019-0007-9.

[13] Zhang Y, Li J, Cheng X, Luo Y, Mai Z, Zhang S. 2018. Community differentiation of bacterioplankton in the epipelagic layer in the South China Sea. Ecol Evol 8:4932–4948. doi:10.1002/ece3.4064. 29876071 PMC5980402

[14] Li L, Lü SH, Cen J. 2019. Spatio-temporal variations of Harmful algal blooms along the coast of Guangdong, Southern China during 1980–2016. J Ocean Limnol 37:535–551. doi:10.1007/s00343-019-8088-y.

[15] Clark MR, Rowden AA, Schlacher T, Williams A, Consalvey M, Stocks KI, Rogers AD, O'Hara TD, White M, Shank TM, Hall-Spencer JM. 2010. The ecology of seamounts: structure, function, and human impacts. Annu Rev Mar Sci 2:253–278. doi:10.1146/annurev-marine-120308-081109. 21141665

[16] Dower J, Mackas D. 1996. “Seamount effects” in the zooplankton community near Cobb Seamount. Deep Sea Res I 43:837–858. doi:10.1016/0967-0637(96)00040-4.

[17] Ma J, Song JM, Li XG, Li N, Wang QD. 2018. Research progress on oceanic seamounts and their eco-environmental characteristics. Mar Sci 42:150–159.

[18] Dai S, Zhao Y, Li X, Wang Z, Zhu M, Liang J, Liu H, Tian Z, Sun X. 2020. The seamount effect on phytoplankton in the tropical western Pacific. Mar Environ Res 162:105094. doi:10.1016/j.marenvres.2020.105094. 32827947

[19] Oliveira AP, Coutinho TP, Cabecadas G, Brogueira MJ, Coca J, Ramos M, Calado G, Duarte P. 2016. Primary production enhancement in a shallow seamount (Gorringe-Northeast Atlantic). J Mar Syst 164:13–29. doi:10.1016/j.jmarsys.2016.07.012.

[20] Bolanos LM, Karp-Boss L, Choi CJ, Worden AZ, Graff JR, Haentjens N, Chase AP, Della Penna A, Gaube P, Morison F, Menden-Deuer S, Westberry TK, O'Malley RT, Boss E, Behrenfeld MJ, Giovannoni SJ. 2020. Small phytoplankton dominate western North Atlantic biomass. ISME J 14:1663–1674. doi:10.1038/s41396-020-0636-0. 32231247 PMC7305139

[21] Djurhuus A, Closek CJ, Kelly RP, Pitz KJ, Michisaki RP, Starks HA, Walz KR, Andruszkiewicz EA, Olesin E, Hubbard K, Montes E, Otis D, Muller-Karger FE, Chavez FP, Boehm AB, Breitbart M. 2020. Environmental DNA reveals seasonal shifts and potential interactions in a marine community. Nat Commun 11:254. doi:10.1038/s41467-019-14105-1. 31937756 PMC6959347

[22] Santoferrara L, Burki F, Filker S, Logares R, Dunthorn M, McManus GB. 2020. Perspectives from ten years of protist studies by high-throughput metabarcoding. J Eukaryot Microbiol 67:612–622. doi:10.1111/jeu.12813. 32498124

[23] Sommeria-Klein G, Watteaux R, Ibarbalz FM, Pierella Karlusich JJ, Iudicone D, Bowler C, Morlon H. 2021. Global drivers of eukaryotic plankton biogeography in the sunlit ocean. Science 374:594–599. doi:10.1126/science.abb3717. 34709919

[24] Kopf A, Bicak M, Kottmann R, Schnetzer J, Kostadinov I, Lehmann K, Fernandez-Guerra A, Jeanthon C, Rahav E, Ullrich M, Wichels A, Gerdts G, Polymenakou P, Kotoulas G, Siam R, Abdallah RZ, Sonnenschein EC, Cariou T, O'Gara F, Jackson S, Orlic S, Steinke M, Busch J, Duarte B, Caçador I, Canning-Clode J, Bobrova O, Marteinsson V, Reynisson E, Loureiro CM, Luna GM, Quero GM, Löscher CR, Kremp A, DeLorenzo ME, Øvreås L, Tolman J, LaRoche J, Penna A, Frischer M, Davis T, Katherine B, Meyer CP, Ramos S, Magalhães C, Jude-Lemeilleur F, Aguirre-Macedo ML, Wang S, Poulton N, Jones S, et al. 2015. The ocean sampling day consortium. Gigascience 4:27. doi:10.1186/s13742-015-0066-5. 26097697 PMC4473829

[25] McNichol J, Berube PM, Biller SJ, Fuhrman JA. 2021. Evaluating and improving small subunit rRNA PCR primer coverage for bacteria, archaea, and eukaryotes using metagenomes from global ocean surveys. mSystems 6:e00565-21. doi:10.1128/mSystems.00565-21. 34060911 PMC8269242

[26] Cui ZM, Xu Q, Gibson K, Liu SY, Chen NS. 2021. Metabarcoding analysis of harmful algal bloom species in the Changjiang Estuary, China. Sci Total Environ 782:146823. doi:10.1016/j.scitotenv.2021.146823.

[27] Huang HL, Xu Q, Gibson K, Chen Y, Chen NS. 2021. Molecular characterization of harmful algal blooms in the Bohai Sea using metabarcoding analysis. Harmful Algae 106:102066. doi:10.1016/j.hal.2021.102066. 34154783

[28] Lin SH, Hu ZX, Deng YY, Shang LX, Gobler CJ, Tang YZ. 2020. An assessment on the intrapopulational and intraindividual genetic diversity in LSU rDNA in the harmful algal blooms-forming dinoflagellate *Margalefidinium* (= *Cochlodinium*) *fulvescens* based on clonal cultures and bloom samples from Jiaozhou Bay, China. Harmful Algae 96:101821. doi:10.1016/j.hal.2020.101821. 32560829

[29] Callahan BJ, McMurdie PJ, Rosen MJ, Han AW, Johnson AJ, Holmes SP. 2016. DADA2: high-resolution sample inference from Illumina amplicon data. Nat Methods 13:581–583. doi:10.1038/nmeth.3869. 27214047 PMC4927377

[30] Callahan BJ, McMurdie PJ, Holmes SP. 2017. Exact sequence variants should replace operational taxonomic units in marker-gene data analysis. ISME J 11:2639–2643. doi:10.1038/ismej.2017.119. 28731476 PMC5702726

[31] Knight R, Vrbanac A, Taylor BC, Aksenov A, Callewaert C, Debelius J, Gonzalez A, Kosciolek T, McCall LI, McDonald D, Melnik AV, Morton JT, Navas J, Quinn RA, Sanders JG, Swafford AD, Thompson LR, Tripathi A, Xu ZJZ, Zaneveld JR, Zhu QY, Caporaso JG, Dorrestein PC. 2018. Best practices for analysing microbiomes. Nat Rev Microbiol 16:410–422. doi:10.1038/s41579-018-0029-9. 29795328

[32] Chen Y, Xu Q, Gibson K, Chen N. 2021. Metabarcoding dissection of harmful algal bloom species in the East China Sea off Southern Zhejiang Province in late spring. Mar Pollut Bull 169:112586. doi:10.1016/j.marpolbul.2021.112586. 34116370

[33] Liu SY, Cui ZM, Zhao YF, Chen NS. 2022. Composition and spatial-temporal dynamics of phytoplankton community shaped by environmental selection and interactions in the Jiaozhou Bay. Water Res 218:118488. doi:10.1016/j.watres.2022.118488. 35489150

[34] Qiao L, Chang Z, Li J, Chen Z. 2020. Phytoplankton community succession in relation to water quality changes in the indoor industrial aquaculture system for *Litopenaeus vannamei*. Aquaculture 527:735441. doi:10.1016/j.aquaculture.2020.735441.

[35] Parada AE, Needham DM, Fuhrman JA. 2016. Every base matters: assessing small subunit rRNA primers for marine microbiomes with mock communities, time series and global field samples. Environ Microbiol 18:1403–1414. doi:10.1111/1462-2920.13023. 26271760

[36] Qiao L, Chang Z, Li J, Chen Z, Yang L, Luo Q. 2019. Phytoplankton community structure and diversity in the indoor industrial aquaculture system for Litopenaeus vannamei revealed by high-throughput sequencing and morphological identification. Aquac Res 50:2563–2576. doi:10.1111/are.14213.

[37] Galluzzi L, Bertozzini E, Penna A, Perini F, Garcés E, Magnani M. 2010. Analysis of rRNA gene content in the Mediterranean dinoflagellate *Alexandrium catenella* and *Alexandrium taylori*: implications for the quantitative real-time PCR-based monitoring methods. J Appl Phycol 22:1–9. doi:10.1007/s10811-009-9411-3.

[38] Xu D, Li R, Hu C, Sun P, Jiao N, Warren A. 2017. Microbial eukaryote diversity and activity in the water column of the South China Sea based on DNA and RNA high throughput sequencing. Front Microbiol 8:1121. doi:10.3389/fmicb.2017.01121. 28659910 PMC5469884

[39] Kunin V, Engelbrektson A, Ochman H, Hugenholtz P. 2010. Wrinkles in the rare biosphere: pyrosequencing errors can lead to artificial inflation of diversity estimates. Environ Microbiol 12:118–123. doi:10.1111/j.1462-2920.2009.02051.x. 19725865

[40] Shokralla S, Spall JL, Gibson JF, Hajibabaei M. 2012. Next generation sequencing technologies for environmental DNA research. Mol Ecol 21:1794–1805. doi:10.1111/j.1365-294X.2012.05538.x. 22486820

[41] Yi X, Zhang D, Sun J, Beardall J, Gao K. 2021. Cyanobacteria-dominated phytoplankton in the oligotrophic South China Sea maintain photosynthetic potential despite diurnal photoinactivation of PSII. Front Mar Sci 8:736586. doi:10.3389/fmars.2021.736586.

[42] Guillou L, Bachar D, Audic S, Bass D, Berney C, Bittner L, Boutte C, Burgaud G, de Vargas C, Decelle J, Del Campo J, Dolan JR, Dunthorn M, Edvardsen B, Holzmann M, Kooistra WHCF, Lara E, Le Bescot N, Logares R, Mahé F, Massana R, Montresor M, Morard R, Not F, Pawlowski J, Probert I, Sauvadet A-L, Siano R, Stoeck T, Vaulot D, Zimmermann P, Christen R. 2013. The Protist Ribosomal Reference database (PR2): a catalog of unicellular eukaryote small sub-unit rRNA sequences with curated taxonomy. Nucleic Acids Res 41:D597–D604. doi:10.1093/nar/gks1160. 23193267 PMC3531120

[43] Mordret S, Piredda R, Vaulot D, Montresor M, Kooistra W, Sarno D. 2018. dinoref: a curated dinoflagellate (Dinophyceae) reference database for the 18S rRNA gene. Mol Ecol Resour 18:974–987. doi:10.1111/1755-0998.12781. 29603631

[44] Quast C, Pruesse E, Yilmaz P, Gerken J, Schweer T, Yarza P, Peplies J, Glockner FO. 2013. The SILVA ribosomal RNA gene database project: improved data processing and web-based tools. Nucleic Acids Res 41:D590–D596. doi:10.1093/nar/gks1219. 23193283 PMC3531112

[45] Tanabe AS, Nagai S, Hida K, Yasuike M, Fujiwara A, Nakamura Y, Takano Y, Katakura S. 2016. Comparative study of the validity of three regions of the 18S-rRNA gene for massively parallel sequencing-based monitoring of the planktonic eukaryote community. Mol Ecol Resour 16:402–414. doi:10.1111/1755-0998.12459. 26309223

[46] Gianna P, Estelle PB, Dominik F, Qu ZS, Bettina S, Thorsten S, Thomas P. 2019. Seasonality of planktonic freshwater ciliates: are analyses based on V9 regions of the 18S rRNA gene correlated with morphospecies counts? Front Microbiol 10:248. doi:10.3389/fmicb.2019.00248. 30837972 PMC6389714

[47] Wu PF, Li DX, Kong LF, Li YY, Zhang H, Xie ZX, Lin L, Wang DZ. 2020. The diversity and biogeography of microeukaryotes in the euphotic zone of the northwestern Pacific Ocean. Sci Total Environ 698:134289. doi:10.1016/j.scitotenv.2019.134289. 31514034

[48] Mouriño B, Fernández E, Serret P, Harbour D, Sinha B, Pingree R. 2001. Variability and seasonality of physical and biological fields at the Great Meteor Tablemount (subtropical NE Atlantic). Oceanologica Acta 24:167–185. doi:10.1016/S0399-1784(00)01138-5.

[49] Arístegui J, Mendonça A, Vilas JC, Espino M, Polo I, Montero MF, Martins A. 2009. Plankton metabolic balance at two North Atlantic seamounts. Deep Sea Res Part II Topical Studies Oceanogr 56:2646–2655. doi:10.1016/j.dsr2.2008.12.025.

[50] Santos M, Moita MT, Bashmachnikov I, Menezes GM, Carmo V, Loureiro CM, Mendonça A, Silva AF, Martins A. 2013. Phytoplankton variability and oceanographic conditions at Condor seamount, Azores (NE Atlantic). Deep Sea Res Part II Topical Studies Oceanogr 98:52–62. doi:10.1016/j.dsr2.2013.05.037.

[51] Genin A, Boehlert GW. 1985. Dynamics of temperature and chlorophyll structures above a seamount: an oceanic experiment. J Mar Res 43:907–924. doi:10.1357/002224085788453868.

[52] Liu L, Wang Z, Lu S. 2020. Diversity and geographical distribution of resting stages of eukaryotic algae in the surface sediments from the southern Chinese coastline based on metabarcoding partial 18S rDNA sequences. Mar Ecol 41:1–17. doi:10.1111/maec.12585.

[53] Xu D, Jiao N, Ren R, Warren A. 2017. Distribution and diversity of microbial eukaryotes in bathypelagic waters of the South China Sea. J Eukaryot Microbiol 64:370–382. doi:10.1111/jeu.12372. 27687286

[54] Xiao W, Wang L, Laws E, Xie Y, Chen J, Liu X, Chen B, Huang B. 2018. Realized niches explain spatial gradients in seasonal abundance of phytoplankton groups in the South China Sea. Progr Oceanogr 162:223–239. doi:10.1016/j.pocean.2018.03.008.

[55] Huang HL, Shao QW, Zhu XJ, Luo J, Meng R, Zhou CX, Zhu P, Zhu YF, Yan XJ. 2019. Distribution of *Karlodinium veneficum* in the coastal region of Xiangshan Bay in the East China Sea, as detected by a real-time quantitative PCR assay of ribosomal ITS sequence. Harmful Algae 81:65–76. doi:10.1016/j.hal.2018.12.001. 30638500

[56] Yang HJ, Hu ZX, Shang LX, Deng YY, Tang YZ. 2020. A strain of the toxic dinoflagellate *Karlodinium veneficum* isolated from the East China Sea is an omnivorous phagotroph. Harmful Algae 93:101775. doi:10.1016/j.hal.2020.101775. 32307067

[57] Agusti S, Gonzalez-Gordillo JI, Vaque D, Estrada M, Cerezo MI, Salazar G, Gasol JM, Duarte CM. 2015. Ubiquitous healthy diatoms in the deep sea confirm deep carbon injection by the biological pump. Nat Commun 6:7608. doi:10.1038/ncomms8608. 26158221 PMC4510647

[58] Song HY, Chen Y, Gibson K, Liu SY, Yu ZM, Chen NS. 2021. High genetic diversity of the harmful algal bloom species *Phaeocystis globosa* revealed using the molecular marker COX1. Harmful Algae 107:102065. doi:10.1016/j.hal.2021.102065. 34456022

[59] Zhang Q, Niu Z, Wang J, Liu C, Kong F, Hu X, Zhao J, Yu R. 2021. Development of high-resolution chloroplast markers for intraspecific phylogeographic studies of *Phaeocystis globose*. J Ocean Limnol 39:508–524. doi:10.1007/s00343-020-9304-5.

[60] Dai X, Lu D, Guan W, Xia P, Wang H, He P, Zhang D. 2013. The correlation between *Prorocentrum donghaiense* blooms and the Taiwan warm current in the East China Sea–evidence for the “Pelagic Seed Bank” hypothesis. PLoS One 8:e64188. doi:10.1371/journal.pone.0064188. 23671709 PMC3650056

[61] Bolyen E, Rideout JR, Dillon MR, Bokulich NA, Abnet CC, Al-Ghalith GA, Alexander H, Alm EJ, Arumugam M, Asnicar F, Bai Y, Bisanz JE, Bittinger K, Brejnrod A, Brislawn CJ, Brown CT, Callahan BJ, Caraballo-Rodríguez AM, Chase J, Cope EK, Da Silva R, Diener C, Dorrestein PC, Douglas GM, Durall DM, Duvallet C, Edwardson CF, Ernst M, Estaki M, Fouquier J, Gauglitz JM, Gibbons SM, Gibson DL, Gonzalez A, Gorlick K, Guo J, Hillmann B, Holmes S, Holste H, Huttenhower C, Huttley GA, Janssen S, Jarmusch AK, Jiang L, Kaehler BD, Kang KB, Keefe CR, Keim P, Kelley ST, Knights D, et al. 2019. Reproducible, interactive, scalable and extensible microbiome data science using QIIME 2. Nat Biotechnol 37:852–857. doi:10.1038/s41587-019-0209-9. 31341288 PMC7015180

[62] Yeh YC, Fuhrman JA. 2022. Contrasting diversity patterns of prokaryotes and protists over time and depth at the San-Pedro Ocean Time series. ISME Commun 2:1–12. doi:10.1038/s43705-022-00121-8. PMC9723720 37938286

[63] De Luca D, Kooistra W, Sarno D, Biffali E, Piredda R. 2021. Empirical evidence for concerted evolution in the 18S rDNA region of the planktonic diatom genus *Chaetoceros*. Sci Rep 11:807. doi:10.1038/s41598-020-80829-6. 33437054 PMC7804092

[64] Leigh JW, Bryant D, Nakagawa S. 2015. popart: full–feature software for haplotype network construction. Methods Ecol Evol 6:1110–1116. doi:10.1111/2041-210X.12410.

[65] Yao YX, Chen NS. 2021. Biodiversity of phytoplankton and red tide species in the Pearl River Estuary. Mar Sci 45:75–90.

[66] Chen NS, Zhang MJ. 2021. Advances in the study of biodiversity of phytoplankton and red tide species in China (III): in the South China Sea. Oceanol Limnol Sin 52:385–401.

