# Supplementary material for: Diversity and Distribution of Harmful Algal Bloom Species from Seamount to Coastal Waters in the South China Sea
Source: Microbiol Spectr. 2023 Feb 23;11(2):e04169-22. doi: 10.1128/spectrum.04169-22 (PMC10100961; doi:10.1128/spectrum.04169-22)
Supplement: Supplemental file 1 — Supplemental material. Download spectrum.04169-22-s0001.pdf, PDF file, 1.4 MB [file spectrum.04169-22-s0001.pdf]

Supplemental Material FOR Figures

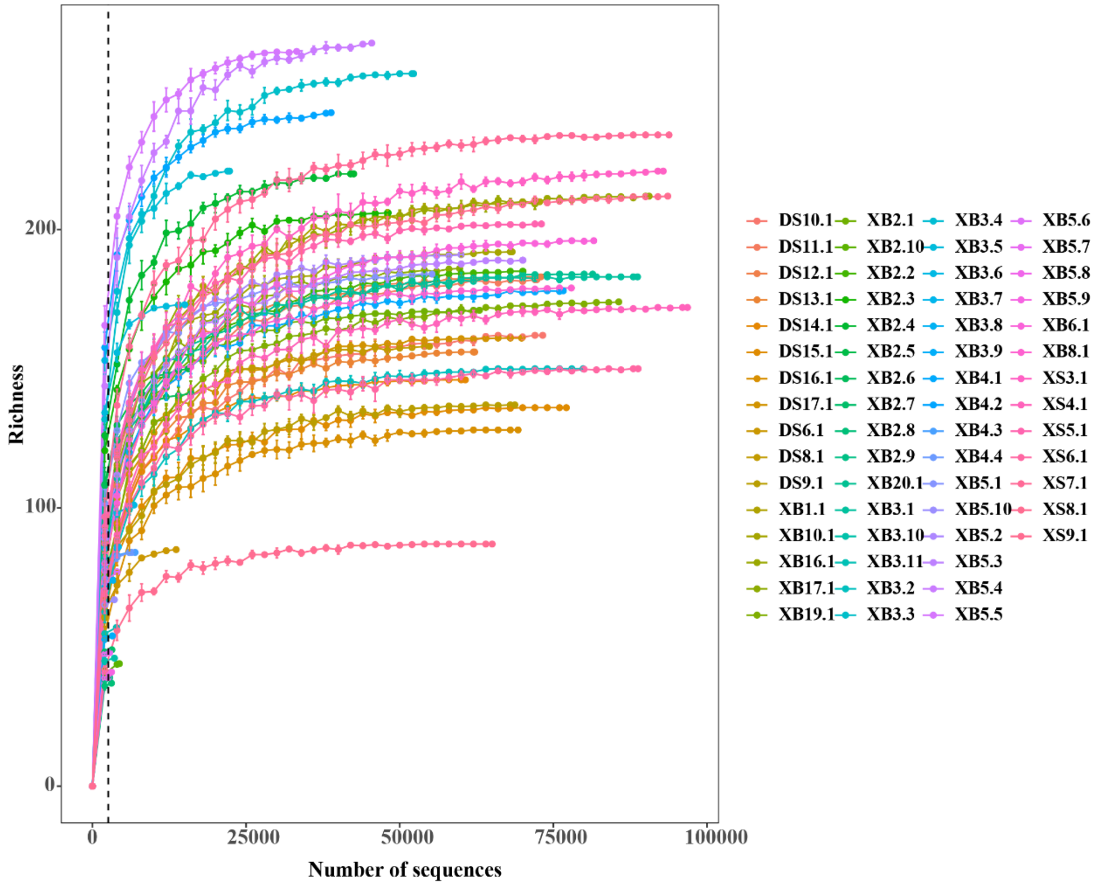

Fig. S1 Rarefaction curves of ASVs. The curves were drawn based on the abundance of reads from 61 samples.

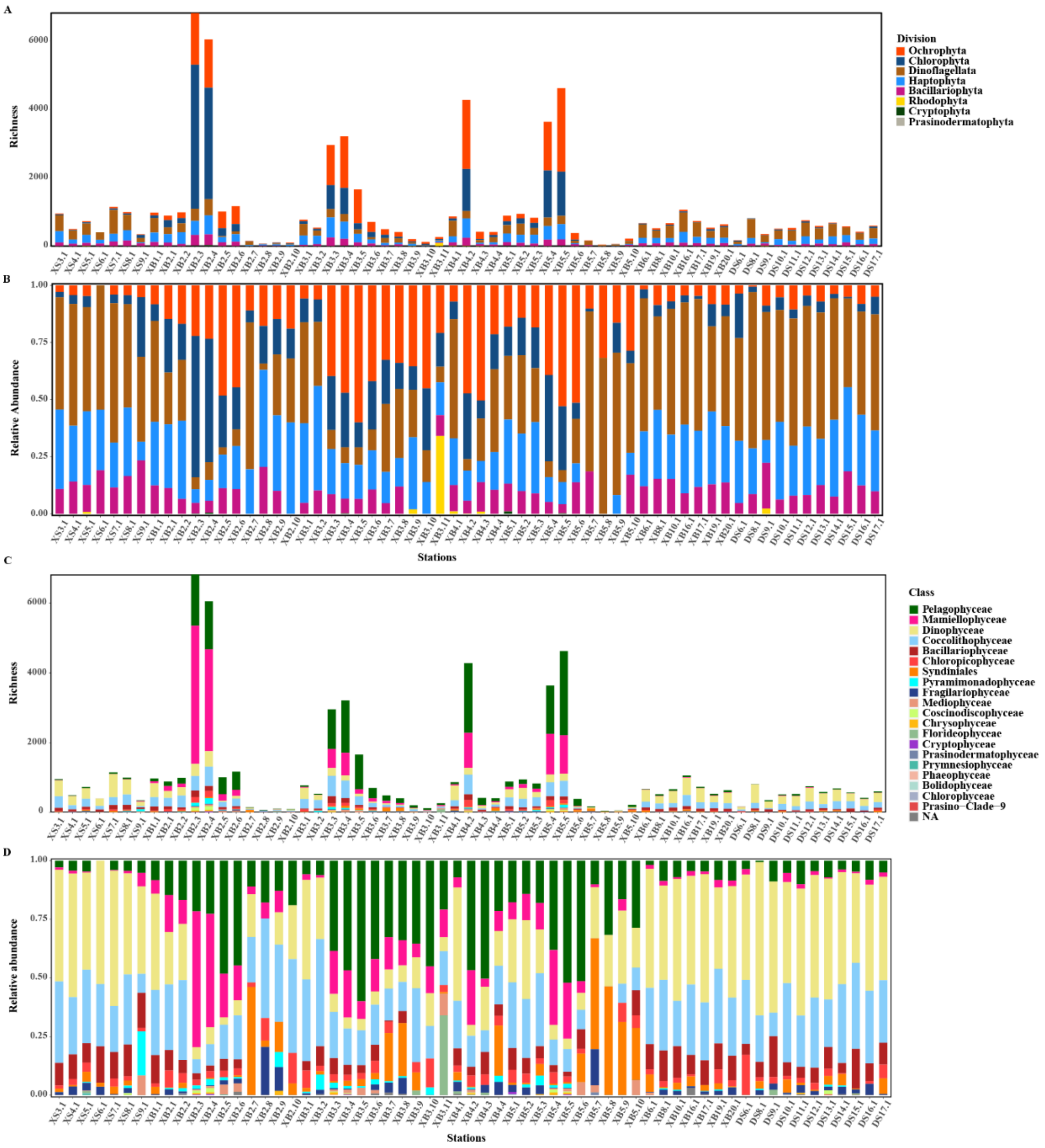

Fig. S2 Taxonomic classification of eukaryotic algae ASVs retrieved from 61 samples. (A) and (B) for richness and relative abundance of eukaryotic algae at division-level taxa; (C) and (D) for richness and relative abundance of eukaryotic algae at class-level taxa.

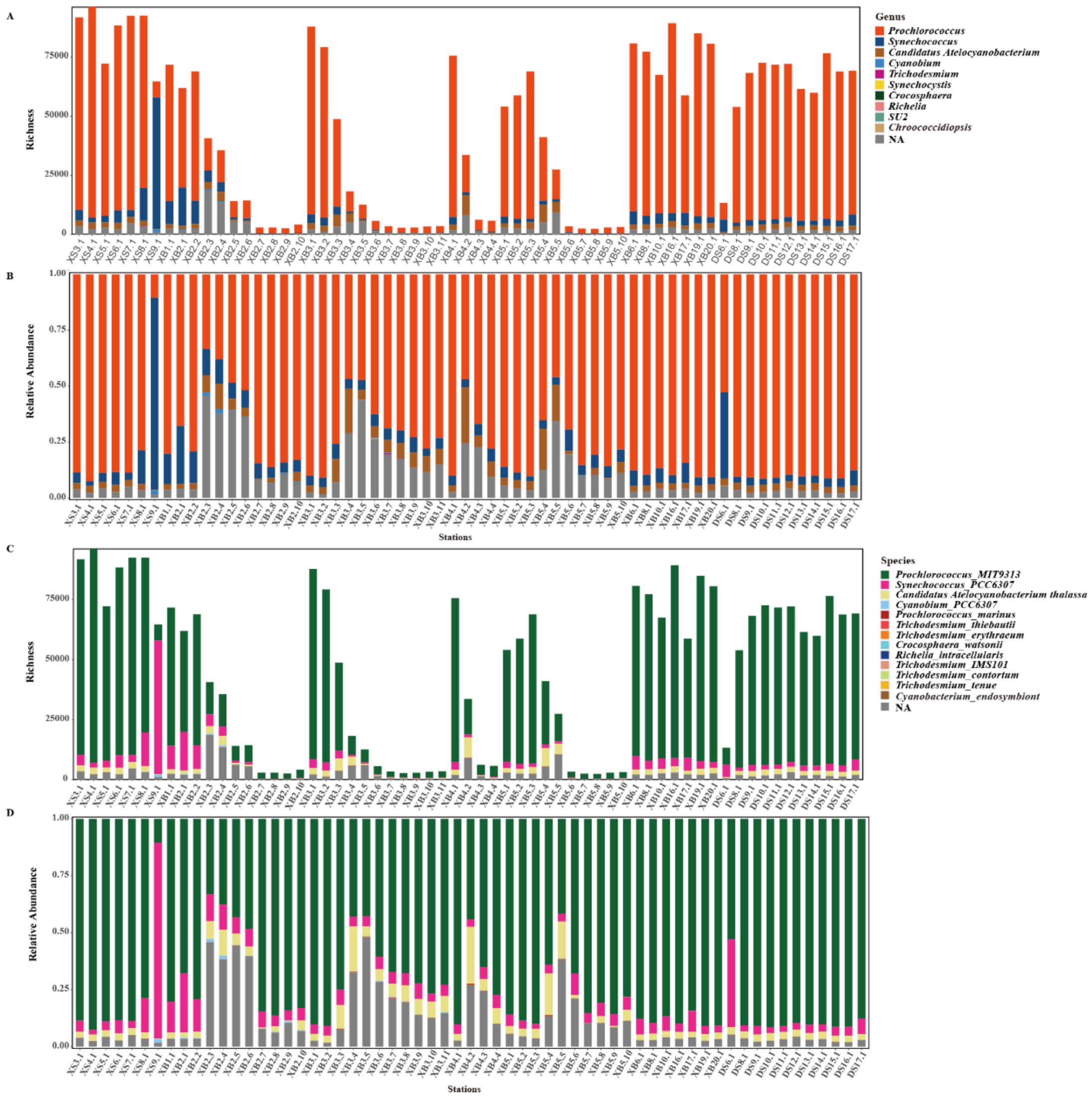

Fig. S3 Taxonomic classification of Cyanobacteria ASVs retrieved from 61 samples. (A) and (B) for richness and relative abundance of Cyanobacteria at Genus-level taxa; (C) and (D) for richness and relative abundance of Cyanobacteria at species-level taxa.

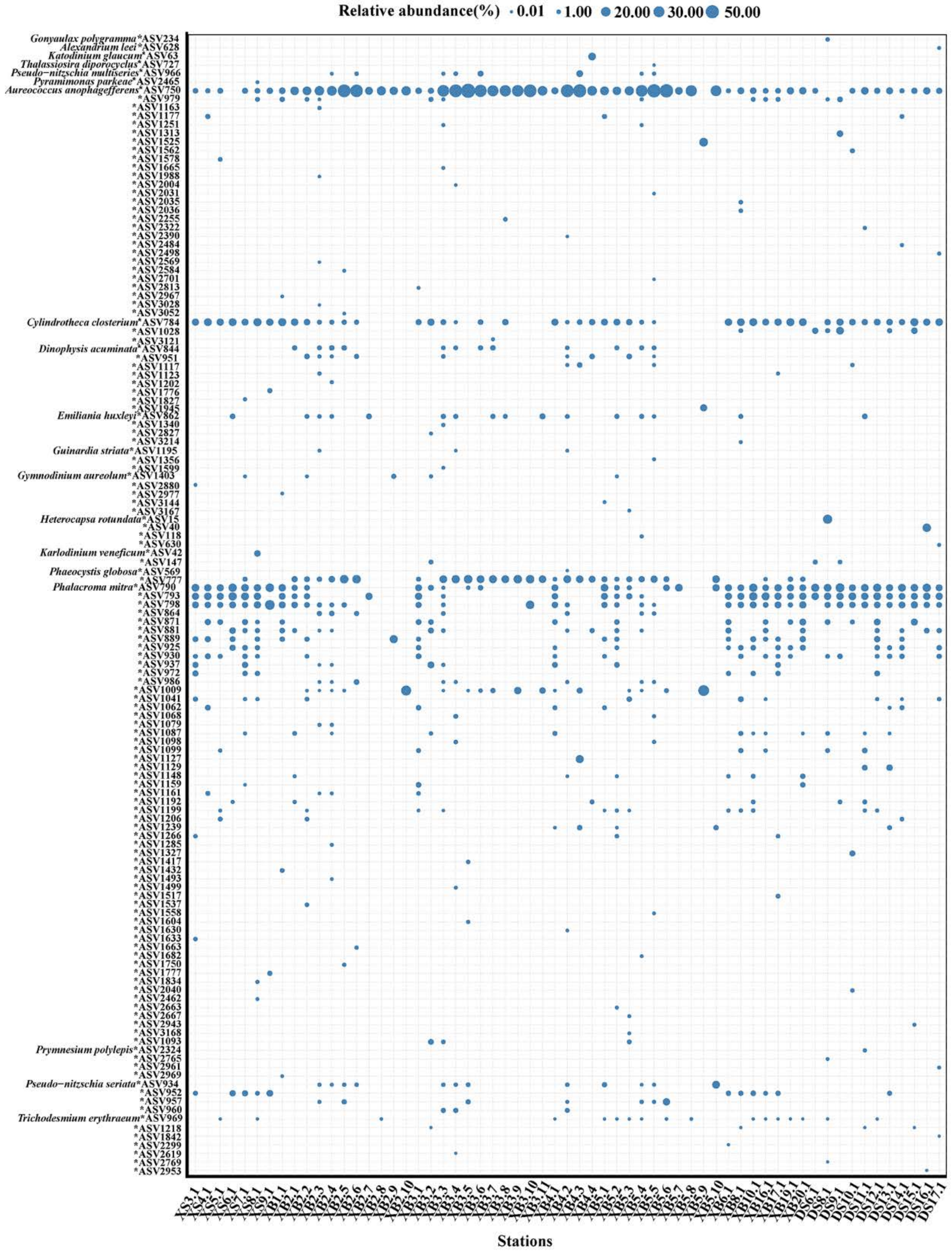

Fig. S4 Distribution of HAB species by one ASV to one species and multiple ASVs to one species from 61 field samples in the South China Sea (SCS). The size of the blue circle represents the size of the relative abundance.

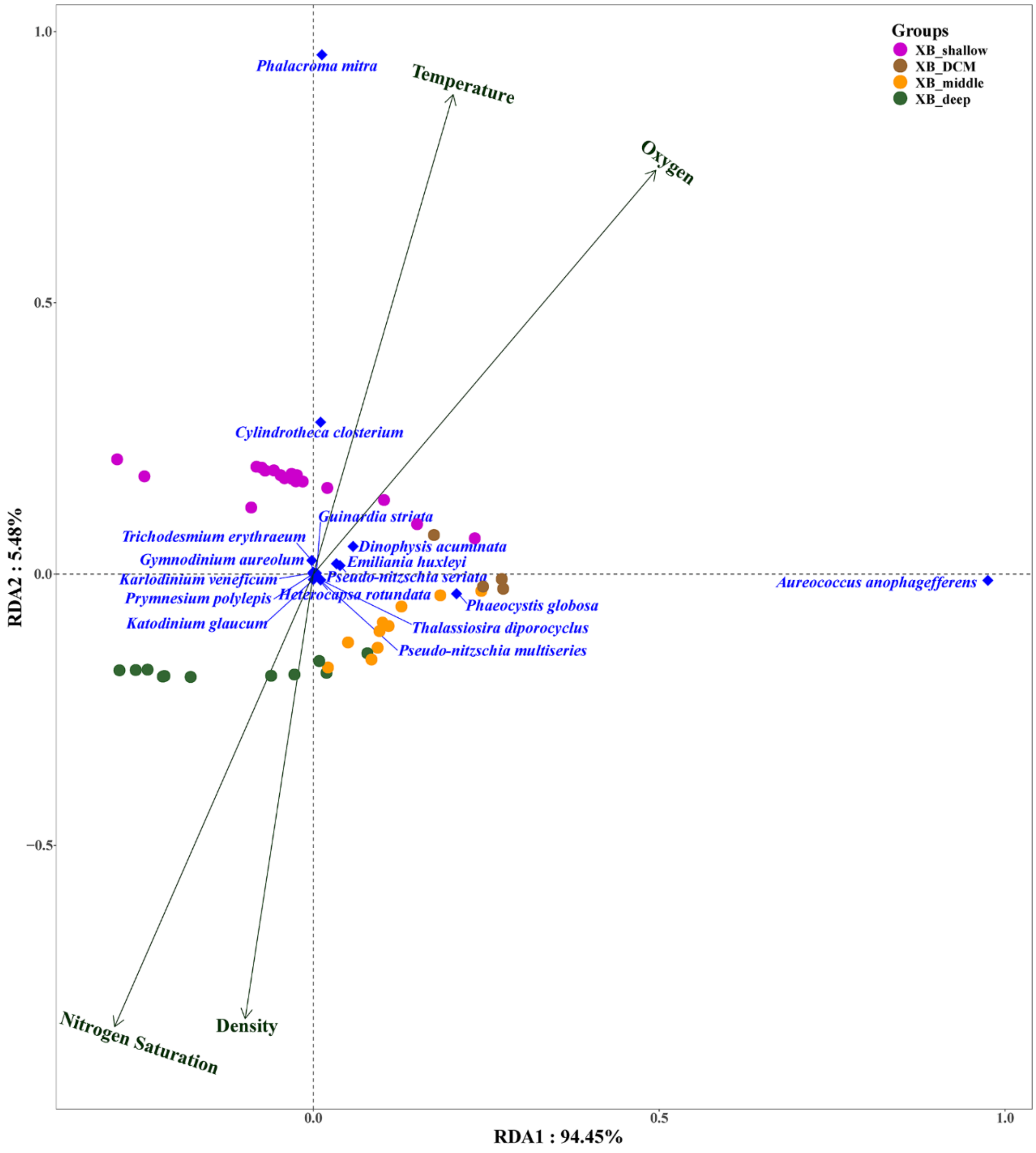

Fig. S5 Major physicochemical parameters in different samples with the depth gradients from seamount region of Xianbei in the South China Sea (SCS).

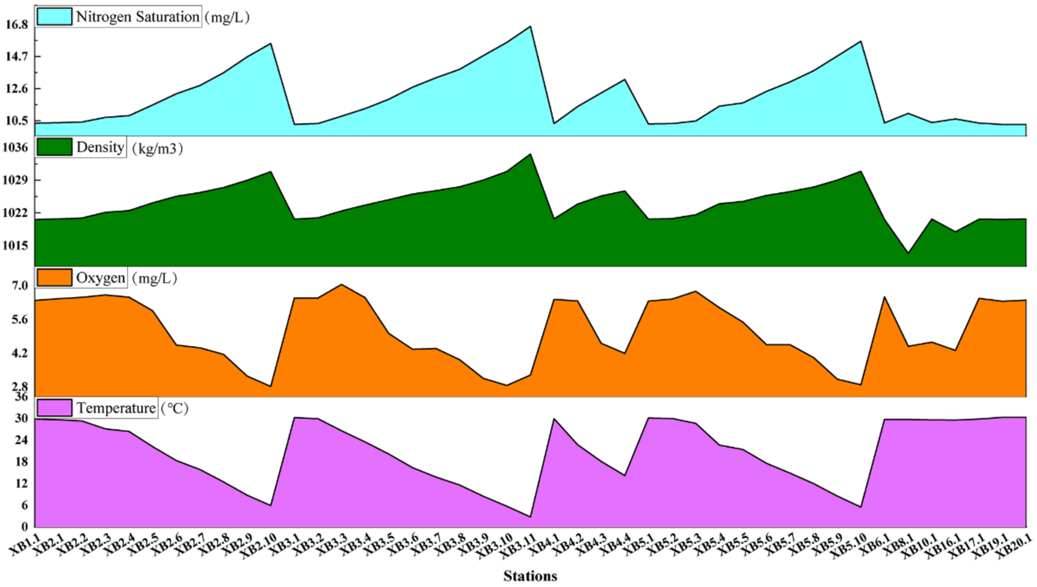

Fig. S6 Correlations between HAB Species and environmental factors in seamount regions of Xianbei. Redundancy analysis (RDA) analysis for testing the relationship between HAB species and environmental factors. Dots in different colors or shapes represent sample groups in different environments or conditions. Species and environmental factors in the RDA diagram are represented by blue and green arrows, respectively.

Supplemental Material FOR Tables

Table S1 The diversity indices for Eukaryotic algae in different samples

Samples	Richness	Shannon	Simpson	Pielou	Chao1	ACE	goods_coverage
XS3.1	35.00	3.125	0.943	0.879	35.000	35.000	1.000
XS4.1	26.00	2.883	0.931	0.885	26.000	26.367	0.998
XS5.1	38.00	3.135	0.942	0.862	38.000	38.422	0.999
XS6.1	19.00	2.625	0.913	0.892	19.000	19.000	1.000
XS7.1	41.00	3.177	0.946	0.856	41.000	41.426	0.999
XS8.1	42.00	3.169	0.941	0.848	42.000	42.000	1.000
XS9.1	12.00	2.239	0.875	0.901	12.000	12.000	1.000
XB1.1	36.00	3.079	0.941	0.859	36.000	36.000	1.000
XB2.1	33.00	3.042	0.937	0.870	33.000	33.000	1.000
XB2.2	42.00	3.181	0.941	0.851	42.200	43.031	0.998
XB2.3	53.00	2.297	0.825	0.578	53.000	53.445	1.000
XB2.4	62.00	2.436	0.835	0.590	62.000	62.420	1.000
XB2.5	21.00	1.850	0.729	0.608	21.000	21.424	0.999
XB2.6	24.00	2.073	0.762	0.652	24.000	24.000	1.000
XB2.7	19.00	2.689	0.918	0.913	19.000	19.000	1.000
XB2.8	7.00	1.549	0.733	0.796	7.000	7.000	1.000
XB2.9	12.00	2.087	0.832	0.840	12.000	12.000	1.000
XB2.10	8.00	1.608	0.744	0.773	8.000	8.000	1.000
XB3.1	37.00	3.267	0.953	0.905	37.000	37.322	0.999
XB3.2	31.00	2.905	0.925	0.846	31.000	31.427	0.998
XB3.3	53.00	2.532	0.821	0.638	53.333	53.781	0.999
XB3.4	48.00	2.043	0.735	0.528	48.000	48.294	1.000
XB3.5	32.00	1.658	0.613	0.478	32.000	32.351	0.999
XB3.6	27.00	2.308	0.795	0.700	27.000	27.000	1.000
XB3.7	31.00	2.549	0.857	0.742	31.000	31.000	1.000
XB3.8	24.00	2.413	0.851	0.759	24.000	24.000	1.000
XB3.9	12.00	1.964	0.806	0.790	12.000	12.000	1.000
XB3.10	7.00	1.605	0.735	0.825	7.000	7.000	1.000
XB3.11	13.00	1.997	0.811	0.779	13.000	13.000	1.000
XB4.1	40.00	3.143	0.936	0.852	40.167	40.979	0.998
XB4.2	40.00	1.965	0.731	0.533	40.000	40.000	1.000
XB4.3	12.00	1.773	0.713	0.713	12.000	12.000	1.000
XB4.4	24.00	2.711	0.908	0.853	24.000	24.000	1.000
XB5.1	37.00	3.124	0.938	0.865	37.000	37.438	0.999
XB5.2	37.00	3.146	0.940	0.871	37.000	37.323	0.999
XB5.3	39.00	2.969	0.924	0.810	39.111	39.861	0.998
XB5.4	52.00	2.317	0.788	0.586	52.000	52.000	1.000
XB5.5	52.00	1.849	0.679	0.468	52.000	52.000	1.000

XB5.6	16.00	1.869	0.711	0.674	16.000	16.000	1.000
XB5.7	14.00	2.340	0.887	0.887	14.000	14.000	1.000
XB5.8	5.00	1.490	0.760	0.926	5.000	5.000	1.000
XB5.9	6.00	1.581	0.763	0.883	6.000	6.000	1.000
XB5.10	16.00	2.370	0.869	0.855	16.000	16.000	1.000
XB6.1	37.00	3.070	0.933	0.850	37.000	37.000	1.000
XB8.1	35.00	3.198	0.949	0.899	35.000	35.000	1.000
XB10.1	35.00	2.972	0.929	0.836	35.000	35.416	0.999
XB16.1	43.00	3.225	0.947	0.857	43.000	43.428	0.999
XB17.1	35.00	3.171	0.946	0.892	35.000	35.000	1.000
XB19.1	26.00	2.931	0.933	0.900	26.000	26.000	1.000
XB20.1	30.00	3.052	0.943	0.897	30.500	30.917	0.997
DS6.1	11.00	2.161	0.864	0.901	11.000	11.000	1.000
DS8.1	39.00	3.054	0.928	0.834	39.000	39.000	1.000
DS9.1	15.00	2.405	0.889	0.888	15.000	15.000	1.000
DS10.1	31.00	3.045	0.941	0.887	31.000	31.000	1.000
DS11.1	34.00	3.041	0.938	0.862	34.000	34.308	0.998
DS12.1	30.00	3.030	0.939	0.891	30.000	30.000	1.000
DS13.1	35.00	3.066	0.933	0.862	35.000	35.361	0.998
DS14.1	38.00	3.118	0.940	0.857	38.000	38.553	0.999
DS15.1	24.00	2.802	0.930	0.882	24.000	24.000	1.000
DS16.1	18.00	2.631	0.917	0.910	18.000	18.000	1.000
DS17.1	38.00	3.067	0.933	0.843	38.000	38.000	1.000

Table S2 The diversity indices for Cyanobacteria in different samples

Samples	Richness	Shannon	Simpson	Pielou	Chao1	ACE	goods_coverage
XS3.1	172.00	1.527	0.540	0.297	172.188	172.916	1.000
XS4.1	138.00	1.281	0.467	0.260	138.125	138.502	1.000
XS5.1	157.00	1.456	0.505	0.288	157.000	157.317	1.000
XS6.1	126.00	1.418	0.518	0.293	126.333	127.016	1.000
XS7.1	183.00	1.510	0.507	0.290	183.000	183.000	1.000
XS8.1	161.00	1.637	0.613	0.322	161.769	162.793	1.000
XS9.1	72.00	1.054	0.393	0.247	72.000	72.000	1.000
XB1.1	162.00	1.661	0.625	0.326	162.214	163.000	1.000
XB2.1	129.00	1.794	0.687	0.369	129.429	129.957	1.000
XB2.2	135.00	1.679	0.625	0.342	135.000	135.340	1.000
XB2.3	143.00	2.535	0.828	0.511	143.000	143.368	1.000
XB2.4	146.00	2.675	0.861	0.537	146.077	146.602	1.000
XB2.5	120.00	3.169	0.911	0.662	120.000	120.000	1.000
XB2.6	110.00	3.083	0.905	0.656	110.000	110.296	1.000
XB2.7	28.00	1.307	0.545	0.392	28.000	28.000	1.000
XB2.8	27.00	1.386	0.556	0.421	27.000	27.000	1.000
XB2.9	27.00	1.346	0.543	0.408	27.000	27.000	1.000
XB2.10	34.00	1.409	0.554	0.400	34.000	34.000	1.000

XB3.1	138.00	1.376	0.496	0.279	138.000	138.000	1.000
XB3.2	113.00	1.410	0.535	0.298	113.000	113.000	1.000
XB3.3	188.00	2.579	0.791	0.493	188.067	188.533	1.000
XB3.4	163.00	3.537	0.945	0.694	163.214	163.975	1.000
XB3.5	132.00	3.372	0.921	0.691	132.000	132.000	1.000
XB3.6	67.00	2.750	0.867	0.654	67.750	68.010	0.999
XB3.7	48.00	2.460	0.813	0.636	48.000	48.000	1.000
XB3.8	49.00	2.362	0.803	0.607	49.000	49.000	1.000
XB3.9	41.00	2.172	0.750	0.585	41.000	41.331	1.000
XB3.10	37.00	1.967	0.700	0.545	37.000	37.000	1.000
XB3.11	42.00	2.147	0.733	0.574	42.000	42.000	1.000
XB4.1	131.00	1.364	0.492	0.280	131.429	131.954	1.000
XB4.2	185.00	3.528	0.943	0.676	186.000	186.962	1.000
XB4.3	67.00	2.656	0.832	0.632	67.000	67.000	1.000
XB4.4	56.00	1.999	0.684	0.497	56.000	56.000	1.000
XB5.1	138.00	1.622	0.543	0.329	138.333	139.103	1.000
XB5.2	141.00	1.486	0.509	0.300	141.063	141.744	1.000
XB5.3	138.00	1.579	0.563	0.320	138.125	138.631	1.000
XB5.4	199.00	3.099	0.886	0.585	199.625	200.879	1.000
XB5.5	194.00	3.672	0.952	0.697	194.000	194.317	1.000
XB5.6	55.00	2.272	0.788	0.567	55.000	55.000	1.000
XB5.7	33.00	1.388	0.534	0.397	33.000	33.000	1.000
XB5.8	32.00	1.494	0.578	0.431	32.000	32.000	1.000
XB5.9	31.00	1.358	0.527	0.395	31.000	31.000	1.000
XB5.10	44.00	1.909	0.652	0.505	44.000	44.000	1.000
XB6.1	150.00	1.503	0.557	0.300	150.059	150.660	1.000
XB8.1	136.00	1.422	0.520	0.290	136.000	136.331	1.000
XB10.1	150.00	1.517	0.542	0.303	150.273	150.933	1.000
XB16.1	159.00	1.429	0.508	0.282	159.375	159.841	1.000
XB17.1	141.00	1.580	0.581	0.319	142.000	143.503	1.000
XB19.1	139.00	1.337	0.482	0.271	139.714	141.092	1.000
XB20.1	146.00	1.373	0.485	0.276	146.000	146.353	1.000
DS6.1	70.00	1.815	0.713	0.427	70.000	70.372	1.000
DS8.1	115.00	1.387	0.502	0.292	115.077	115.719	1.000
DS9.1	118.00	1.341	0.506	0.281	118.000	118.345	1.000
DS10.1	122.00	1.372	0.510	0.286	122.769	123.677	1.000
DS11.1	142.00	1.380	0.492	0.278	142.125	142.515	1.000
DS12.1	145.00	1.405	0.486	0.282	145.000	145.285	1.000
DS13.1	116.00	1.356	0.493	0.285	116.083	116.791	1.000
DS14.1	103.00	1.417	0.506	0.306	103.000	103.000	1.000
DS15.1	108.00	1.227	0.452	0.262	108.000	108.286	1.000
DS16.1	104.00	1.216	0.453	0.262	104.000	104.327	1.000
DS17.1	117.00	1.420	0.525	0.298	117.000	117.000	1.000

Table S3 Major physicochemical parameters in different sampling stations from seamount region of Xianbei

Stations	Depth (m)	Longitude (°E)	Latitude (°N)	Temperature (℃)	Oxygen (mg/L)	Density (Kg/m^3)	Nitrogen Saturation (mg/L)
XB1.1	-5	116.83	16.89	29.79	6.38	1020.58	10.34
XB2.1	-5	116.79	16.77	29.64	6.46	1020.71	10.36
XB2.2	-25	116.79	16.77	29.3	6.52	1020.9	10.41
XB2.3	-50	116.79	16.77	27.11	6.62	1022.12	10.71
XB2.4	-60	116.79	16.77	26.36	6.52	1022.48	10.82
XB2.5	-100	116.79	16.77	22.18	5.96	1024.15	11.52
XB2.6	-150	116.79	16.77	18.32	4.54	1025.55	12.26
XB2.7	-200	116.79	16.77	15.85	4.42	1026.35	12.8
XB2.8	-300	116.79	16.77	12.44	4.15	1027.44	13.64
XB2.9	-500	116.79	16.77	8.75	3.25	1028.98	14.68
XB2.10	-800	116.79	16.77	5.92	2.82	1030.83	15.56
XB3.1	-5	116.75	16.69	30.19	6.49	1020.69	10.26
XB3.2	-25	116.75	16.69	29.89	6.48	1020.92	10.3
XB3.3	-50	116.75	16.69	26.57	7.05	1022.41	10.79
XB3.4	-75	116.75	16.69	23.48	6.5	1023.68	11.29
XB3.5	-100	116.75	16.69	20.15	5.02	1024.81	11.9
XB3.6	-150	116.75	16.69	16.38	4.37	1026.02	12.68
XB3.7	-200	116.75	16.69	13.74	4.39	1026.76	13.31
XB3.8	-300	116.75	16.69	11.59	3.93	1027.59	13.87
XB3.9	-500	116.75	16.69	8.46	3.15	1029.03	14.76
XB3.10	-800	116.75	16.69	5.73	2.86	1030.86	15.63
XB3.11	-1500	116.75	16.69	2.77	3.3	1034.57	16.68
XB4.1	-5	116.72	16.62	29.92	6.43	1020.7	10.31
XB4.2	-80	116.72	16.62	22.73	6.36	1023.88	11.42
XB4.3	-150	116.72	16.62	18.08	4.61	1025.62	12.31
XB4.4	-200	116.72	16.62	14.17	4.19	1026.68	13.2
XB5.1	-5	116.69	16.55	30.1	6.36	1020.68	10.28
XB5.2	-25	116.69	16.55	29.97	6.44	1020.8	10.3
XB5.3	-50	116.69	16.55	28.62	6.77	1021.62	10.48
XB5.4	-90	116.69	16.55	22.61	6.09	1023.96	11.44
XB5.5	-100	116.69	16.55	21.4	5.48	1024.45	11.66
XB5.6	-150	116.69	16.55	17.59	4.55	1025.74	12.42
XB5.7	-200	116.69	16.55	14.85	4.55	1026.56	13.04
XB5.8	-300	116.69	16.55	11.95	4.02	1027.52	13.77
XB5.9	-500	116.69	16.55	8.53	3.12	1029.01	14.74
XB5.10	-800	116.69	16.55	5.49	2.89	1030.9	15.71
XB6.1	-5	116.65	16.47	29.73	6.53	1020.58	10.35
XB8.1	-5	116.57	16.32	29.73	4.49	1013.35	10.97

XB10.1	-5	116.48	16.17	29.57	4.66	1020.68	10.37
XB16.1	-5	117.02	16.58	29.51	4.31	1017.94	10.61
XB17.1	-5	116.87	16.62	29.79	6.47	1020.64	10.34
XB19.1	-5	116.65	16.68	30.31	6.35	1020.61	10.25
XB20.1	-5	116.27	16.8	30.28	6.4	1020.67	10.25
